# Beech tree masting explains the inter-annual variation in the fall and spring peaks of *Ixodes ricinus* ticks with different time lags

**DOI:** 10.1186/s13071-021-05076-8

**Published:** 2021-11-08

**Authors:** Cindy Bregnard, Olivier Rais, Coralie Herrmann, Olaf Kahl, Katharina Brugger, Maarten J. Voordouw

**Affiliations:** 1 Laboratory of Ecology and Evolution of Parasites, Institute of Biology, University of Neuchâtel, Neuchâtel, Switzerland; 2 Laboratory of Ecology and Epidemiology of Parasites, Institute of Biology, University of Neuchâtel, Neuchâtel, Switzerland; 3 Laboratory of Eco-Epidemiology of Parasites, Institute of Biology, University of Neuchâtel, Neuchâtel, Switzerland; 4 tick-radar GmbH, 10555 Berlin, Germany; 5 Unit for Veterinary Public Health and Epidemiology, University of Veterinary Medicine Vienna, Veterinärplatz 1, 1210 Vienna, Austria; 6 Department of Veterinary Microbiology, Western College of Veterinary Medicine, University of Saskatchewan, Saskatoon, Canada

**Keywords:** Beech tree, Bimodal phenology, Climate, Diapause, *Ixodes ricinus*, *Fagus sylvatica*, Masting, Tick population ecology, Time lag, Unimodal phenology

## Abstract

**Background:**

The tick *Ixodes ricinus* is an important vector of tick-borne diseases including Lyme borreliosis. In continental Europe, the nymphal stage of *I. ricinus* often has a bimodal phenology with a large spring peak and a smaller fall peak. There is consensus about the origin of the spring nymphal peak, but there are two alternative hypotheses for the fall nymphal peak. In the direct development hypothesis, larvae quest as nymphs in the fall of the same year that they obtained their larval blood meal. In the developmental diapause hypothesis, larvae overwinter in the engorged state and quest as nymphs one year after they obtained their larval blood meal. These two hypotheses make different predictions about the time lags that separate the larval blood meal and the density of questing nymphs (DON) in the spring and fall.

**Methods:**

Inter-annual variation in seed production (masting) by deciduous trees is a time-lagged index for the density of vertebrate hosts (e.g., rodents) which provide blood meals for larval ticks. We used a long-term data set on the masting of the European beech tree and a 15-year study on the DON at 4 different elevation sites in western Switzerland to differentiate between the two alternative hypotheses for the origin of the fall nymphal peak.

**Results:**

Questing *I. ricinus* nymphs had a bimodal phenology at the three lower elevation sites, but a unimodal phenology at the top elevation site. At the lower elevation sites, the DON in the fall was strongly correlated with the DON in the spring of the following year. The inter-annual variation in the densities of *I. ricinus* nymphs in the fall and spring was best explained by a 1-year versus a 2-year time lag with the beech tree masting index. Fall nymphs had higher fat content than spring nymphs indicating that they were younger. All these observations are consistent with the direct development hypothesis for the fall peak of *I. ricinus* nymphs at our study site. Our study provides new insight into the complex bimodal phenology of this important disease vector.

**Conclusions:**

Public health officials in Europe should be aware that following a strong mast year, the DON will increase 1 year later in the fall and 2 years later in the spring. Studies of *I. ricinus* populations with a bimodal phenology should consider that the spring and fall peak in the same calendar year represent different generations of ticks.

**Graphical Abstract:**

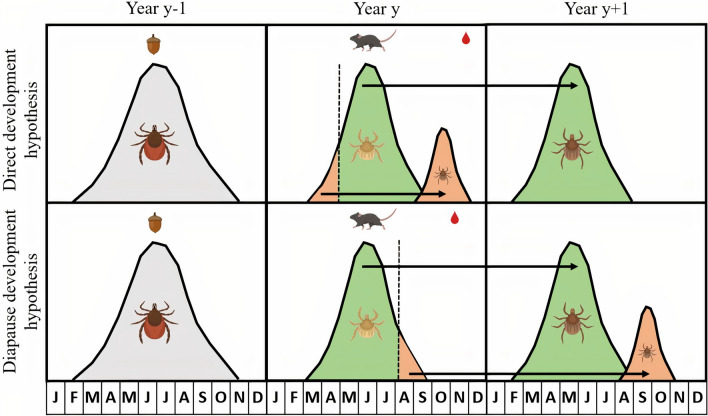

**Supplementary Information:**

The online version contains supplementary material available at 10.1186/s13071-021-05076-8.

## Background

The incidence of tick-borne diseases is increasing in Europe and North America [1–7]. In large parts of Europe, the hard tick *Ixodes ricinus* is an important vector of tick-borne diseases including Lyme borreliosis and tick-borne encephalitis [8, 9]. This tick species consists of three motile stages, larva, nymph, and adult, that must obtain a blood meal from a vertebrate host to moult into the next stage (or produce eggs in the case of adult female ticks). The population ecology of *I. ricinus* is complicated by several factors. First, *I. ricinus* can feed on a wide variety of vertebrate hosts (e.g., lizards, birds, Eulipotyphla, rodents, carnivores, and ungulates) for which the population density is often unknown [10, 11]. Second, the life cycle takes 3 to 6 years to complete [12–14], which introduces time lags [15–17]. For example, the density of nymphs in year *y* depends on the feeding success of the larvae in year *y* − 1, which in turn depends on the ratio of larvae to vertebrate hosts in year *y* − 1 [18, 19]. Third, the existence of diapause as an adaptation to surviving cold winters can split the same cohort of ticks into groups that are active at different times of the year [12, 20, 21]. Uncertainty about the origin of these groups complicates our ability to model the underlying ecological factors and appropriate time lags that drive inter-annual variation in tick abundance.

Long-term field studies have shown that a combination of abiotic and biotic factors drive inter-annual variation in tick abundance. *Ixodes* ticks spend more than 99% of their life cycle off the host, where they are exposed to changes in temperature and precipitation [20, 22]. The life history traits of *Ixodes* ticks, such as development rates and survival rates, are highly sensitive to temperature and relative humidity [20, 23–25]. Tick population ecology is also highly sensitive to the abundance of vertebrate hosts because all tick stages must blood-feed to graduate to the next stage in the life cycle [26, 27]. Small mammals (e.g., rodents) are an important but variable source of food for immature ticks (larvae and nymphs); rodent populations often exhibit inter-annual fluctuations due to variation in their food supply [28–34]. Studies on *I. ricinus* in Europe and on *I. scapularis* in North America have shown that inter-annual variation in seed production (also known as masting) by deciduous trees drives inter-annual variation in the density of nymphs 2 years later; this relationship is mediated by rodents that feed on the tree seeds and provide blood meals for the larvae [15, 17, 18, 35–39]. In summary, masting in the fall of year *y* − 2 enhances rodent density and larval feeding success in the spring and summer of year *y* − 1, which increases the density of nymphs in year *y*.

*Ixodes ricinus* has a distinct seasonal activity pattern (phenology) that allows them to search for vertebrate hosts (a behaviour called questing) when abiotic conditions (e.g., temperature and humidity) are favourable. Diapause is a critical adaptation that allows ticks (and other arthropods) to overwinter in an inactive state and thereby avoid physiological damage caused by cold winter temperatures. Behavioural diapause is the suppression of host-seeking activity by unfed ticks in the fall in anticipation of unfavourable winter conditions. Developmental diapause is the cessation of development by blood-engorged ticks in the fall to enhance overwinter survival [40]. Both types of diapause are driven by changes in photoperiod (the most reliable predictor of seasonal change) and both are important for structuring the phenology of *I. ricinus* ticks [21, 23], which varies widely among geographic locations [12, 20, 21, 41, 42]. In some parts of Europe, nymphs and adult ticks exhibit a unimodal phenology where questing activity peaks in late spring or early summer and ends in the fall (Table 1) [43–47]. In central Europe, the most common phenology is bimodal with a large peak of activity in spring/early summer and a smaller peak of activity in fall (Table 1) [21].

**Table 1:** Type of phenology for *Ixodes ricinus* ticks in different countries in Europe

Phenology	Country	Region	Studies
Unimodal	United Kingdom	Powys	[43]
Unimodal	Sweden	Bogesund	[44]
Unimodal	Switzerland	Chaumont Mountain	[45, 46]
Unimodal	Netherlands		[47]
Bimodal	Ireland	County Wicklow	[48]
Bimodal	Sweden	Bogesund	[44]
Bimodal	Russia	Crimea	[41]
Bimodal	Switzerland	Chaumont Mountain, Salins	[45, 46, 49–52]
Bimodal	United Kingdom	Exmoor National Park, Mynydd Mallaen, Dorset	[53]
Bimodal	Spain	Rioja region	[54]
Bimodal	Czech Republic	Prague	[55]
Bimodal	Finland	Seili, Jyväskylä	[56, 57]
Bimodal	Germany	Haselmuehl	[58]
Bimodal	Austria	Vienna	[59]

*Ixodes ricinus* ticks (nymphs and adults) have either a unimodal or bimodal phenology. The phenology is shown for different countries and regions in Europe. The decision of whether the phenology was unimodal or bimodal in Table 1 was based on whether the authors of the study identified the phenology as either unimodal or bimodal in the article

**Fig. 1:**
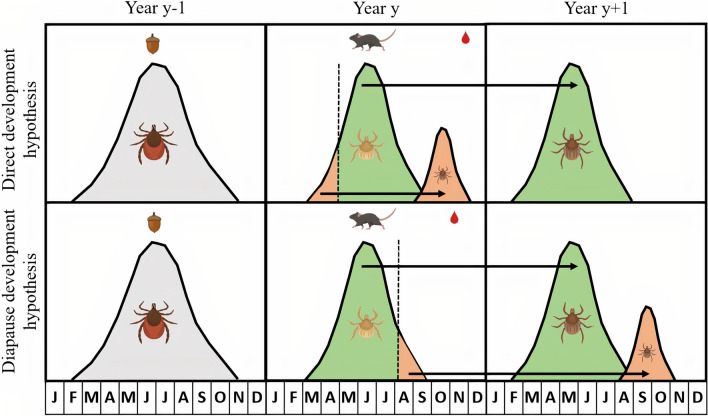
Two alternative (but not mutually exclusive) hypotheses for the fall peak are shown: the direct development hypothesis (top panel) and the developmental diapause hypothesis (bottom panel). For simplicity, we show a fast 3-year life cycle of *I. ricinus*, which includes adult ticks (year *y* − 1 in the left panel) that lay eggs, the larvae (year *y* in the middle panel), and the nymphs (year *y* + 1 in the right panel). Masting occurs in year *y* − 1 (represented by the acorn symbol), the rodent population increases in year *y* (represented by the mouse symbol), and the larval blood meal occurs in year *y* (represented by the blood drop symbol). The origin of the spring peak of nymphs (green peak in year *y* + 1) is the same for both hypotheses and is as follows. Larvae that obtain their blood meal in the summer (green fraction in year *y*) moult into unfed nymphs in the same year, enter behavioural diapause, overwinter, and quest as unfed nymphs the following spring (green peak in year *y* + 1). The two hypotheses differ with respect to the origin of the fall nymphal peak (small orange peak). In the direct development hypothesis, larvae that obtain their blood meal early in the summer (orange fraction in year *y*) moult into unfed nymphs in summer and quest as unfed nymphs that same fall (small orange peak in year *y*). In the developmental diapause hypothesis, larvae that obtain their blood meal late in the summer (orange fraction in year *y*) overwinter as engorged larvae, moult into unfed nymphs the following summer, and quest as unfed nymphs that fall (small orange peak in year *y* + 1). The time lag between a masting event (acorns in year *y* − 1) and the spring peak of nymphs (green peak in year *y* + 1) is 2 years. In contrast, the time lag between a masting event (acorns in year *y* − 1) and the fall peak of nymphs (small orange peak) is 1 year under the direct development hypothesis and 2 years under the developmental diapause hypothesis

Two alternative explanations for this bimodal phenology of questing *I. ricinus* nymphs are the developmental diapause hypothesis and the direct development hypothesis (Fig. 1) [21, 41, 45, 46, 53, 60, 61]. The developmental diapause hypothesis (Fig. 1) suggests that the seasonal timing of the larval blood meal splits the larval cohort into two groups of nymphs that are active in the spring and fall of the following year [12, 21]. Larvae that obtain their blood meal in early summer moult into unfed nymphs, enter behavioural diapause in fall, overwinter as unfed nymphs, and begin questing the following spring. In contrast, larvae that obtain their blood meal in late summer or early fall enter developmental diapause, overwinter as engorged larvae, complete their development the following summer, and quest that fall (i.e., 1 year after the larval blood meal) [21]. The direct development hypothesis (Fig. 1) suggests that the seasonal timing of the larval blood meal splits the larval cohort into two groups of nymphs that are active in the fall of that year and the spring of the following year [12, 21]. Larvae that obtain their blood meal in early summer moult into unfed nymphs and quest that same fall (i.e. a few months after the larval blood meal). In contrast, larvae that obtain their blood meal in late summer or early fall moult into unfed nymphs, enter behavioural diapause, overwinter as unfed nymphs, and quest the following spring [21]. In both hypotheses, there is a 1-year time lag between larval feeding and the spring nymphal peak. The critical distinction between these two hypotheses is the time lag between larval feeding and the fall nymphal peak, which is 1 year for the developmental diapause hypothesis and a few months for the direct development hypothesis. To date, it is not clear which of these two hypotheses is more important for explaining the fall peak of *I. ricinus* nymphs.

In addition to influencing tick life history traits (development, survival, and reproduction) and seasonal phenology, the weather also influences tick questing behaviour, which in turn determines the probability that ticks are captured by common tick sampling methods (e.g., dragging). Thus, while the seasonal phenology in central Europe dictates that questing nymphs are most abundant in spring and early summer, the questing activity of nymphs on any given day depends on the weather [23, 49, 62]. Field plot experiments have shown that the percentage of ticks that are actively questing depends on the weather [42, 50]. Questing activity is generally determined by water balance regulation, which is affected by both temperature and relative humidity [63–65]. In summary, variation in the abundance of questing ticks at any given time depends on three separate mechanisms: (1) time-lagged ecological factors that influence tick life history traits, (2) photoperiod-dependent diapause that determines the broad seasonal activity patterns of questing ticks, and (3) daily weather conditions interacting with the tick water balance that determine whether or not ticks will quest on that day. Separating these three mechanisms, which operate on different temporal scales, is not an easy task.

We previously used a long-term data set (15 years) on the abundance of questing *I. ricinus* ticks at four different elevations on a mountain in western Switzerland to investigate the ecological factors that influence the inter-annual variation in the density of questing nymphs (DON) and the density of questing nymphs infected with the causative agents of Lyme borreliosis (DIN) [15, 39]. The most important finding in these two studies was that inter-annual variation in the DON and the DIN was strongly associated with inter-annual variation in the production of seeds by European beech trees 2 years prior. For these two studies, we analysed the annual abundance of nymphs for the calendar year (1 January to 31 December). This approach assumes that the spring and fall peaks of questing nymphs in the same calendar year are both governed by the same ecological factors and time lags. This assumption may be correct for the developmental diapause hypothesis but it is incorrect for the direct development hypothesis. Another limitation of our previous study was that we investigated a highly limited set of climate variables calculated as annual means for either the current year or the previous year. In contrast, numerous studies suggest that season rather than calendar year is the relevant time scale over which climate variables influence the population ecology of *Ixodes* ticks [16, 66]. By dividing the calendar year into different seasons, we are increasing the temporal resolution at which our climate variables can explain inter-annual variation in tick abundance.

In the present study, we build on our previous modelling efforts of the same data set to investigate three objectives: first, determine whether the developmental diapause hypothesis or the direct development hypothesis is better at explaining inter-annual variation in the fall peak of *I. ricinus* nymphs; second, determine whether seasonal climate means are better than annual climate means at predicting inter-annual variation in the density of nymphs, and which seasonal climate variables are important; third, determine whether we can use generalized additive models (GAMs) to model the complex bimodal seasonal phenology of *I. ricinus* nymphs and whether this approach yields additional insights into the factors that explain seasonal variation in questing tick abundance.

## Methods

### Study location

**Fig. 2:**
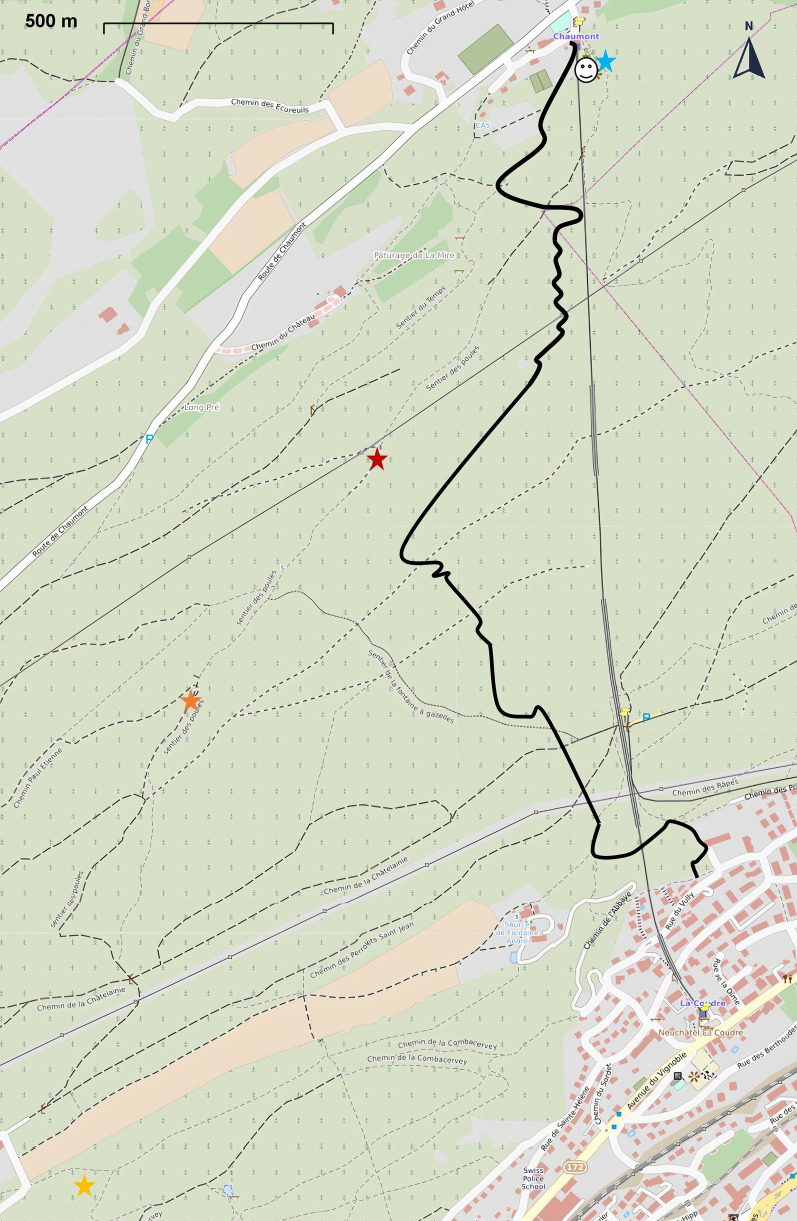
Map of the four elevation sites on Chaumont Mountain, canton of Neuchâtel, Switzerland. The stars represent the four different elevation sites: low elevation site (yellow star; 47° 00′ 15.5″ N 6° 56′ 33.9″ E), medium elevation site (orange star; 47° 00′ 50.8″ N 6° 56′ 51.7″ E), high elevation site (red star; 47° 01′ 10.1″ N 6° 57′ 12.9″ E), and top elevation site (blue star; 47° 01′ 34.6″N 6° 57′ 29.2″E). The smiley represents the outdoor adventure park, and the solid black line represents the mountain biking trail

The study was conducted on the south-facing slope of Chaumont Mountain, which is part of the Jura mountains, and is in the canton of Neuchâtel, in western Switzerland. Four tick sampling sites, referred to as low, medium, high, and top, were established at elevations of 620, 740, 900, and 1073 m above sea level (ASL), respectively, and have been described previously (Fig. 2) [46, 51]. There is logging in the area, and there are hiking trails and recreation areas for the public. The forest on Chaumont Mountain is composed mainly of European beech (*Fagus sylvatica*; 28.6%), Norway spruce (*Picea abies*; 28.5%), European silver fir (*Abies alba*; 20.4%), sycamore maple (*Acer pseudoplatanus*; 5.9%), European ash (*Fraxinus excelsior*; 3.7%), Scots pine (*Pinus sylvestris*; 2.3%), sessile oak (*Quercus petraea*; 2.3%), willow (*Salix* spp.; 2.1%), common whitebeam (*Sorbus aria*; 1.6%), and European hornbeam (*Carpinus betulus*; 1.0%) [67]. The European beech is the most important tree species for this study because we have detailed information on its masting history at our study location (see below). The relative abundance of this tree species is similar between the bottom (31.0% at 600 m ASL) and the top (25.0% at 1000 m ASL) of Chaumont Mountain (J. Boni, forestry engineer of Neuchâtel, personal communication, 5 July 2021).

### Sampling questing *I. ricinus* ticks in the field

Questing *I. ricinus* nymphs and adult ticks were collected monthly over a period of 15 years (January 2004 to December 2018) at each of the four elevation sites. Questing *I. ricinus* larvae were encountered, but they were not counted or collected. The sampling protocol has been described previously [51]. Briefly, a 1-m^2^ cotton flag was dragged over the ground along a fixed transect with a length of 100 m (low elevation) or 120 m (other elevations). The flag was inspected every 20 m, and nymphs and adult ticks were counted separately and placed in collection vials. This method of tick collection targets questing ticks and removes them from the environment; these removed ticks cannot be encountered on future sampling occasions, and they cannot contribute to future tick population growth. The same person (Olivier Rais) conducted all 720 transects (4 elevations × 15 years × 12 months = 720 transects). No dragging was performed on days when there was snow on the ground (hereafter referred to as snow days). Over the study period, a total of 34 snow days occurred, which resulted in missing data for 136 transects.

### Field-measured climate variables

Temperature (units are °C) and relative humidity (RH; units are %) were recorded at 60 cm above ground at one moment in time on the day of tick sampling (usually between 10:00 a.m. and 2:00 p.m.) at each tick sampling site using a thermohygrometer (testo 615, Testo SA, Lonay, Switzerland). Thus, for each combination of elevation and year, we had a total of 12 field measurements of temperature and RH. The saturation deficit (SD) is a measure of the drying power of the atmosphere (units are mm of mercury) and is calculated using temperature (*T*; units are °C) and RH (units are %) as follows: SD = (1 − RH/100) × 4.9463 × e^0.0621×*T*^ [45, 68]. The accuracy of our field-measured climate data was confirmed by comparing them to weather station data [15].

### Weather station climate variables

We also obtained climate data from the CLIMAP-net database of the Federal Office of Meteorology and Climatology MeteoSwiss. Two weather stations close to our study site are in Neuchâtel at 485 m ASL (WMO number = 06604) and in Chaumont at 1136 m ASL (WMO number = 06608). These weather stations sample the temperature and RH every hour, and the total precipitation each day at 200 cm above ground. We used the daily mean temperature (average of the 24 measurements per day), the daily mean RH (average of the 24 measurements per day), and the daily total precipitation (rain, snow). Thus, for each year and each weather station, we had a total of 365 measurements of these three climate variables. The SD was calculated as previously described. For each of the four elevation sites, we calculated site-specific climate variables by interpolating the data between the two weather stations (Additional file 1: Section S1).

### Data on inter-annual variation in tree masting

We previously demonstrated that the abundance of *I. ricinus* ticks depends on the seed production of deciduous trees [15, 39]. The seeds or fruits of forest trees (e.g., acorns of oak trees or beech nuts of beech trees) are often referred to as mast. The annual production of mast by a population of trees in an area occurs in the fall and is highly variable among years [69]. The MASTREE database contains data on masting (or seed production) for many locations in Europe from 1982 to 2016 for two tree species, European beech (*Fagus sylvatica*) and Norway spruce (*Picea abies*) [70]; these two species account for 57.1% of the trees at our study location. In the MASTREE database, the mast intensity is classified into five classes, 1, 2, 3, 4, and 5, which refer to very poor mast, poor mast, moderate mast, good mast, and full mast, respectively [70]. We used this database [70] to obtain masting data for the European beech for the canton of Neuchâtel for the years of our study (2004–2018). We excluded Norway spruce from the analysis because our previous work found that their mast scores 2 years prior were not associated with the inter-annual variation in the DON or the DIN [15, 39].

### Fat content of *I. ricinus* nymphs collected in the spring and fall

Fat is a non-renewable source of energy derived from each blood meal that ticks use to quest for hosts and to maintain their water balance [68, 71, 72]. As *I. ricinus* feeds once per life stage and has no other energy sources between blood meals, their fat content is a physiological index of their current age and future longevity in the unfed state [43, 53, 68]. In a previous study on the *I. ricinus* population at our field site, we collected nymphs in the spring and fall of 2010 and measured their fat content [73]. In the present study, we compared the fat content between spring and fall nymphs. The direct development hypothesis predicts that the fall nymphs are younger and should therefore have a higher fat content compared to the spring nymphs (~ 3 months versus ~ 9 months since the larval blood meal). In contrast, the developmental diapause hypothesis predicts that the fall nymphs are older and should therefore have a lower fat content compared to the spring nymphs (~ 9 months versus ~ 12 months).

## Statistical methods

The MASTREE database contains data on masting from 1982 to 2016, whereas our tick surveillance study ran from January 2004 to December 2018. We therefore had the beech masting scores 1 year prior for the monthly values of the DON in 2017 but not for the DON in 2018. For this reason, the statistical analyses are restricted to a 14-year period (2004 to 2017).

### The density of nymphs (DON)

The density of nymphs (DON) is a measure of the monthly abundance of questing nymphs per 100 m^2^. Over the 14-year study period that was covered by the MASTREE database, estimates of the DON were obtained from 558 fixed monthly transects (4 elevations × 14 years × 12 months = 672 transects; 114 missing transects due to snow and other reasons; 672 transects − 114 transects = 558 transects). The transects on the snow days were coded as missing data.

### Definition of the spring nymphal peak and the fall nymphal peak

Previous work on the abundance of questing *I. ricinus* nymphs at Chaumont Mountain and at other nearby sites have shown a bimodal phenology, with a large peak of the DON in the spring and a smaller peak of the DON in the fall [45, 46, 49–51]. To test which variables best explain inter-annual variation in the spring and fall peaks of the DON, a date must be chosen to separate these two groups of nymphs. We decided that the spring peak included the nymphs sampled from 1 January to 31 August, whereas the fall peak included the nymphs sampled from 1 September to 31 December. These cut-off dates for spring and fall (31 August and 1 September) were chosen because the DON reached a minimum at this time.

### Annual beech masting score

Previous studies have shown that there is a 2-year time lag between masting events and the annual DON and the annual DIN [15, 17, 38, 39]. Our recent analyses of the same data showed that inter-annual variation in the DON and the DIN was strongly associated with the mast scores 2 years prior of European beech but not Norway spruce [15, 39]. Upon further reflection, we realized that while the 2-year time lag is true for the spring peak, it may not be true for the fall peak. The developmental diapause hypothesis and the direct development hypothesis predict that the time lag between the beech masting index and the fall peak of nymphs should be 2 years versus 1 year, respectively. To test these two hypotheses, we created three different explanatory variables, BM[2,2], BM[1,1], and BM[2,1], for the beech masting (BM) index. BM[2,2] assumes there is a 2-year time lag between BM and the spring and fall peaks of nymphs. BM[1,1] assumes there is a 1-year time lag between BM and the spring and fall peaks of nymphs. BM[2,1] assumes there is a 2-year time lag and a 1-year time lag between BM and the spring and fall peaks, respectively. BM[2,2] is consistent with the developmental diapause hypothesis, BM[2,1] is consistent with the direct development hypothesis, and BM[1,1] is not consistent with either hypothesis.

### Weather on the day of tick sampling

To investigate the possible influence of weather on nymphal questing activity, we used the field-measured weather data on the day of tick sampling. An important advantage of the field-measured data compared to the weather station data was that they are specific for each of the four elevation sites. We did not use the field-measured weather data to calculate annual or seasonal means because there were not enough data (i.e., only 12 measurements per calendar year at each site).

### Annual and seasonal mean climate variables

**Fig. 3:**
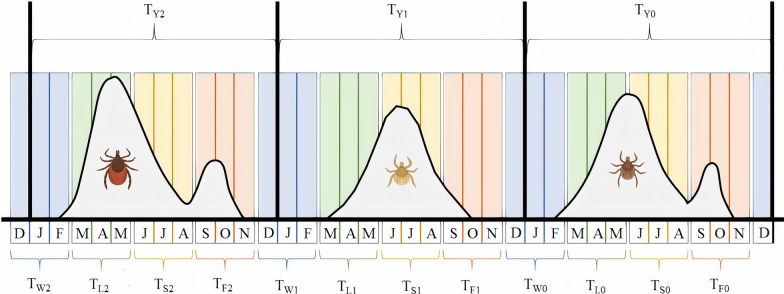
Visual representation of how the mean annual climate variables and the mean seasonal climate variables were calculated over three different years. The approximate 3-year life cycle of *I. ricinus* is shown by adult ticks that lay eggs (left panel), the eggs that hatch into larvae (middle panel), and the larvae that become nymphs (right panel). The bimodal or unimodal distribution in each panel represents the phenology of each tick stage. The three calendar years are shown by the vertical black lines; the months are shown by the bars that are labelled below the X-axis. The four seasons of winter, spring, summer, and fall are colour-coded as blue, green, yellow, and orange, respectively. The response variable of interest is the density of nymphs (DON) in the right panel. For illustrative purposes, the explanatory variable is temperature (abbreviated as *T*). The three annual means of the temperature are *T*_Y2_, *T*_Y1_, and *T*_Y0_ (labelled at the top); the subscript ‘Y’ indicates that the temperature is averaged over the calendar year; the subscripts 0, 1, and 2 indicate the time lag (i.e., 0, 1, or 2 years before the year of the DON). There are 12 seasonal means of the temperature (labelled at the bottom); the subscripts ‘W’, ‘L’, ‘S’, and ‘F’ indicate that the temperature is averaged over the winter, spring (lent), summer, and fall; the subscripts 0, 1, and 2 indicate the time lag in years. For example, *T*_W2_ is the mean temperature of the winter 2 years before the year of the DON

Tick life history traits (development, survival, and reproduction) depend on abiotic factors such as temperature, RH, SD, and precipitation (rain, snow). A great unknown is the relevant time frame over which these abiotic climate variables operate on tick life history traits. For example, the DON in the spring might depend on the climate conditions of the previous winter (e.g., overwinter survival of unfed nymphs) or on the climate conditions of the previous summer, which would influence the rates at which larvae obtain and digest their blood meals and moult into nymphs. In our previous studies on inter-annual variation in the DON and DIN [15, 39], we calculated annual means for the climate variables that were based on a 12-month calendar year. However, many steps in the tick life cycle happen over shorter time scales; for example, engorged larvae take ~ 6 to 8 weeks to moult into nymphs at room temperature [74]. Thus, tick life history traits may depend on climate variables that are operating over shorter temporal windows (e.g., seasons rather than years). We therefore calculated mean seasonal climate variables for each of the 12 seasons (3 years × 4 seasons per year = 12 seasons) that preceded and encompassed the year of tick sampling (Fig. 3). The seasonal means for the winter, spring, summer, and fall were calculated as follows: 1 December (e.g., previous year) to 28/29 February, 1 March to 31 May, 1 June to 31 August, and 1 September to 30 November. As time lags are important in tick ecology, we calculated our annual mean climate variables and seasonal mean climate variables in the present year, the previous year, or 2 years prior (Fig. 3). Thus, for each climate variable, there were a total of 12 seasonal means and 3 annual means. The exception was the annual snowfall (units of cm), which was calculated over the time interval from 1 October (e.g., previous year) to 31 May.

### The monthly DON was analysed using generalized additive models (GAMs)

We used generalized additive models (GAMs) with extra-binomial errors to model the non-linear bimodal seasonal phenology of the monthly DON, which represent count data. GAMs are like generalized linear models, but an important difference is that smoother functions are used to model the response variable as a non-linear function of one or more explanatory variables. The smoother functions are non-parametric (they are like moving average functions) and can be used to fit any complex curve, and this flexibility is a great strength of the GAM approach. A weakness is that there are no parameter estimates for the explanatory variables that are modelled with the smoother functions, which makes model interpretation more difficult. In our preliminary analyses, we found that a site-specific smoother function of the covariate calendar day (where 1 January and 31 December correspond to day 1 and day 365) was the best at capturing the bimodal phenology of the DON over the calendar year.

### Explanatory variables used to model the DON in the base model

We performed model selection in two steps. In the first step, we compared the two alternative hypotheses for the fall peak: the direct development hypothesis and the developmental diapause hypothesis. We used our previous work on the same data set as starting point [15, 39]. The response variable was the monthly DON per 100 m^2^ and rounded to the nearest integer. The explanatory variables included nymphal peak (two levels: spring versus fall), elevation site (four levels: low, medium, high, top), beech masting index (covariate; range = 1 to 5), and year (covariate; rescaled as 1, 2, 3, …, 14). As previously discussed, there were three different beech masting indices: BM[2,2], BM[1,1], and BM[2,1]. We included an interaction between site and year, as our previous work had shown that this interaction was important [15, 39]. We also tested for interactions between nymphal peak, elevation site, and beech masting. To model the bimodal phenology of the DON over the calendar year, we included a site-specific smoother function of the calendar day (covariate; rescaled as 1, 2, 3, …, 365). In the second step, we identified the most important climate variables (see below).

### AIC-based model selection of the base model

We used model selection based on the corrected Akaike information criterion (AICc) to select the best model. Models were ranked according to their AICc values; the best model was the one that had the fewest number of parameters and that was within one unit of the model with the lowest AICc value [75]. We used the difference in AICc values between the models to calculate the Akaike weights, which indicate the percent support, using all the models in the set [76]. For the best model, we determined the statistical significance of the explanatory variables in the fixed effects structure using Wald tests, and we present their parameter estimates. We assessed the goodness of fit for the best model (Additional file 1: Section S2).

### Identification of climate variables

The best model identified in the first step was used as a base model in the second step. In the second step, we investigated the effects of 66 climate variables on the monthly DON (Table 2). The climate variables included the 3 field-measured climate variables of temperature (t), relative humidity (rh), and saturation deficit (sd) on the day of tick sampling. We also included the mean annual and mean seasonal climate variables obtained from the weather stations for temperature (T), RH, SD, precipitation (PR), and annual snowfall (SN). For four climate variables (T, RH, SD, PR), there were 3 annual means and 12 seasonal means; for SN there were 3 annual means.

**Table 2:** Acronym and definition of each variable used in the present study

Acronym	Description
DON	Monthly density of nymphs per 100 m^2^
S	Site name (factor with 4 levels: low, medium, high, top)
Y	Year of the study (covariate: 1, 2, …, 15)
D	Day of the year for the tick sampling (covariate: 1, 2, …, 365)
BM_[1,1]_	Beech mast index in year *y* − 1 (covariate: 1, 2, …, 5)
BM_[2,2]_	Beech mast index in year *y* − 2 (covariate: 1, 2, …, 5)
BM_[2,1]_	Beech mast index with a 2-year time lag for the spring peak and a 1-year time lag for the fall peak (covariate: 1, 2, …, 5)
t	Temperature on day of tick sampling from the field-collected data (°C)
rh	Relative humidity on day of tick sampling from the field-collected data (%)
sd	Saturation deficit on day of tick sampling from the field-collected data (mmHg)
T_Y[*i*]_	Mean annual temperature in year [*i*] from the weather station data (°C); the subscript [*i*] refers to one of three different years: *y*, *y* − 1, and *y* − 2, which are denoted as 0, 1, and 2, respectively
RH_Y[*i*]_	Mean annual relative humidity in year [*i*] from the weather station data (%); the subscript [*i*] refers to one of three different years: *y*, *y* − 1, and *y* − 2, which are denoted as 0, 1, and 2, respectively
SD_Y[*i*]_	Mean annual saturation deficit in year [*i*] from the weather station data (mmHg); the subscript [*i*] refers to one of three different years: *y*, *y* − 1, and *y* − 2, which are denoted as 0, 1, and 2, respectively
PR_Y[*i*]_	Mean annual precipitation in year [*i*] from the weather station data (mm); the subscript [*i*] refers to one of three different years: *y*, *y* − 1, and *y* − 2, which are denoted as 0, 1, and 2, respectively
SN_Y[*i*]_	Annual snowfall in year [*i*] from the weather station data (cm); the subscript [*i*] refers to one of three different years: *y*, *y* − 1, and *y* − 2, which are denoted as 0, 1, and 2, respectively. This variable is calculated from 1 October (e.g., previous year) to 31 May
T_[*x*, *i*]_	Mean seasonal temperature of season [*x*] in year [*i*] from the weather station data (°C); the subscript [x] refers to four different seasons: spring (L), summer (S), fall (F), winter (W); the subscript [*i*] refers to one of three different years: *y*, *y* − 1, and *y* − 2, which are denoted as 0, 1, and 2, respectively
RH_[*x*, *i*]_	Mean seasonal relative humidity of the fall in year *y* from the weather station data (%); the subscript [*x*] refers to four different seasons: spring (L), summer (S), fall (F), winter (W); the subscript [*i*] refers to one of three different years: *y*, *y* − 1, and *y* − 2, which are denoted as 0, 1, and 2, respectively
SD_[*x*, *i*]_	Mean seasonal saturation deficit of the fall in year *y* from the weather station data (mmHg); the subscript [*x*] refers to four different seasons: spring (L), summer (S), fall (F), winter (W); the subscript [*i*] refers to one of three different years: *y*, *y* − 1, and *y* − 2, which are denoted as 0, 1, and 2, respectively
PR_[*x*, *i*]_	Mean seasonal precipitation of the winter in year *y* from the weather station data (mm); the subscript [*x*] refers to four different seasons: spring (L), summer (S), fall (F), winter (W); the subscript [*i*] refers to one of three different years: *y*, *y* − 1, and *y* − 2, which are denoted as 0, 1, and 2, respectively

For 66 climate variables (Table 2), the set of possible models to investigate is very large (2^69^ = 5.902958e+20 models). We therefore decided to include a maximum of two climate variables. To determine which climate variables were important, we created 66 models, which consisted of the base model (identified in the previous step) and one of the 66 climate variables. We then updated the base model by including the best climate variable and then repeated this process for a second time. To allow for maximum flexibility of the relationship between the DON and the climate variables, we modelled the climate variables using smoother functions that included interactions with elevation site or nymphal peak. As before, we used AIC-based model selection to identify the best model.

### Count data and extra-binomial errors

As the DON represents count data (number of nymphs per 100 m^2^), we initially used the Poisson distribution to model the residual errors. The residuals from these models were highly overdispersed; the ratio of the residual deviance (7025) to the residual degrees of freedom (500) was > 1 (7025/500 = 14.05). This problem of overdispersion was solved by using the negative binomial distribution to model the residual errors; the residuals from these models were no longer overdispersed (517/514 = 1.01). Parameter estimates from models with Poisson errors (or extra-binomial errors) are reported on a log scale. Thus, the exponential function must be applied to the parameter estimates to calculate the predicted values of the DON on the original scale.

Models with Poisson errors (or extra-binomial errors) analyse count data, which are integers. However, the monthly DONs were not always integers, because they had been measured over a transect area of 120 m^2^ before being standardized to an area of 100 m^2^. These non-integer DON values had to be rounded to the nearest integer to run GAMs with Poisson errors (or extra-binomial errors).

### Predicted values of the DON

To determine the effect of the explanatory variables on the DON, we used our GAMs to calculate the predicted values. Demonstrating the effect of an explanatory variable of interest requires that the other explanatory variables be set to defined reference conditions. Unless otherwise specified, the reference conditions were as follows: site was low elevation, year was 2004, and the BM[2,1] was 1, calendar dates for spring and fall were 15 April and 15 October, respectively. For simplicity, we present the parameter estimates from the top model rather than the model-averaged parameter estimates.

### Statistical software

We used R version 4.0.3 for all statistical analyses [77]. We used the *gam()* function and the *anova.gam()* function in the mgcv package to run the GAMs and the Wald tests of statistical significance [78]. We used the *mod.sel()* function in the MuMIn package to create the model selection tables [76]. We used the *ggeffect()* function in the ggeffects package to calculate the predicted values of the DON for the GAMs. We used the *ggplot()* function in the ggplot2 package to create the graphs that show the predicted values of the DON for the explanatory variables in the GAMs [79].

## Results

### Mean monthly DON at each of the four elevation sites

The mean monthly DON was inversely related to the altitudinal gradient; it was highest at the low elevation site and lowest at the top elevation site, and these differences were significant (ANOVA: *F*_(3, 554)_ = 102.34, *P* < 0.0001; Table 3; Additional file 1: Section S3). The mean monthly DON at the low, medium, high, and top elevation sites was 74.4, 61.4, 42.7, and 10.6 nymphs per 100 m^2^, respectively (Table 3). If the low elevation site was set as the reference, the mean monthly DON at the medium, high, and top elevation sites were 17.5%, 42.6%, and 85.8% lower, respectively (Table 3). These estimates of the mean DON do not consider the effects of any other explanatory variables.

**Table 3:** Monthly density of nymphs (DON) per 100 m^2^ for the four elevation sites on Chaumont Mountain over the 15 years of the study (2004–2018)

Site	Years	*N*	DON Mean	DON StdDev	DON Range	CND	DON2
Low	15	144	74.4	82.6	0.00–430.0	22,629	62.0
Medium	15	144	61.4	75.0	0.00–385.8	18,849	51.6
High	15	137	42.7	52.0	0.00–257.5	12,582	34.5
Top	15	133	10.6	14.5	0.00–80.8	3000	8.2

Shown for each of the four elevation sites are the number of years, sample size (*N*; units are number of transects), mean, standard deviation, and the range of the monthly DON (units are nymphs per 100 m^2^). For each elevation site, the expected sample size is 180 transects; missing transects are due to snow days when dragging for ticks was not possible. The DON is biased high due to the missing values for the snow days. In our previous study [15], we calculated the cumulative nymphal density (CND) for each calendar year by integrating the area under the curve of the seasonal phenology of the DON (per 100 m^2^) from 1 January to 31 December. When this CND is divided by 365 days, it gives a second estimate of the density of nymphs (DON2) that is less biased by the missing snow days. For this reason, the estimates of DON2 are lower than the DON

### *Ixodes ricinus* nymphs on Chaumont Mountain have a bimodal phenology

**Fig. 4:**
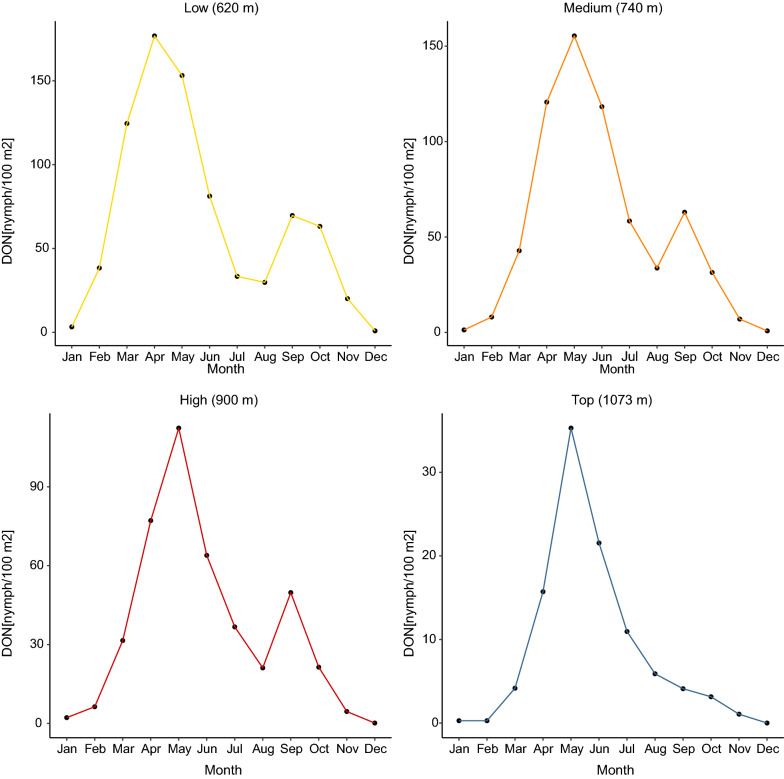
Seasonal changes in the DON over the calendar year at the four elevation sites. The DON is an estimate of the number of questing *I. ricinus* nymphs per 100 m^2^ sampled by the dragging method each month. For each month, the data are averaged over the 15 years of the study (2004–2018). A bimodal phenology with a large peak of the DON in the spring and a smaller peak of the DON in the fall was observed at the low, medium, and high elevation sites, but not at the top elevation site, where the unimodal phenology was characterized by a single peak in the spring

The seasonal changes in the DON over the calendar year at the four elevation sites clearly showed a bimodal phenology, with a large peak of the DON in the spring and a smaller peak of the DON in the fall (Fig. 4). This bimodal phenology of the questing nymphs was observed at the low, medium, and high elevation sites, but not at the top elevation site, where the phenology was characterized by a single peak in the spring. For each of the four elevation sites, we compared the size of the spring and fall nymphal peaks by calculating the area under the curve of the seasonal phenology (Table 4). Expressed as a percent of the total, the fall peak at the low, medium, high, and top elevation sites was 14.9%, 11.5%, 12.5%, and 5.5%, respectively. Thus, the fall peak was largest for the low site and smallest for the top site. Interestingly, the spring peak occurred in April for the low site whereas it occurred in May at the medium, high, and top elevation sites.

**Table 4:** Mean size of the spring and fall peaks of *I. ricinus* nymphs for the four elevation sites on Chaumont Mountain

Site	Cumulative spring peak (CSP)					
	*N*	CSP mean	CSP StdDev	CSP range	CND2	CSP (%)
Low	15	18,145	10,508	7516–50,801	21,033	85.1
Medium	15	15,573	8538	7247–35,767	17,293	88.5
High	15	10,191	6300	3284–24,780	11,357	87.5
Top	15	2693	1416	842–4974	2826	94.5

Site	Cumulative fall peak (CFP)					
	*N*	CFP mean	CFP StdDev	CFP range	CND2	CFP (%)
Low	15	2887	2456	507–9462	21,033	14.9
Medium	15	1720	1303	308–4361	17,293	11.5
High	15	1166	925	249–2763	11,357	12.5
Top	15	134	129	14–399	2826	5.5

The size of the spring peak and the fall peak of *I. ricinus* nymphs are shown for each of the four elevation sites on Chaumont Mountain. To compare the size of the cumulative spring peak (CSP) and the cumulative fall peak (CFP), we integrated the area under the curve of the seasonal phenology of the DON (per 100 m^2^) from 1 January to 31 August (CSP), and from 1 September to 31 December (CFP), respectively. The interpretation of the CSP and CFP are the numbers of *I. ricinus* nymphs that would have been captured if we had sampled for ticks every day over the corresponding calendar dates. For the CSP and the CFP, the sample size (*N* = 15 years), mean, standard deviation (StdDev), and range are shown. A second estimate of the cumulative nymphal density (CND2) was calculated by summing the CSP and the CFP. To express the two peaks as a percentage, the CSP and the CFP were each divided by the CND2

### Correlation between fall peak in year *y* - 1 and the spring peak in year y supports the direct development hypothesis

**Fig. 5:**
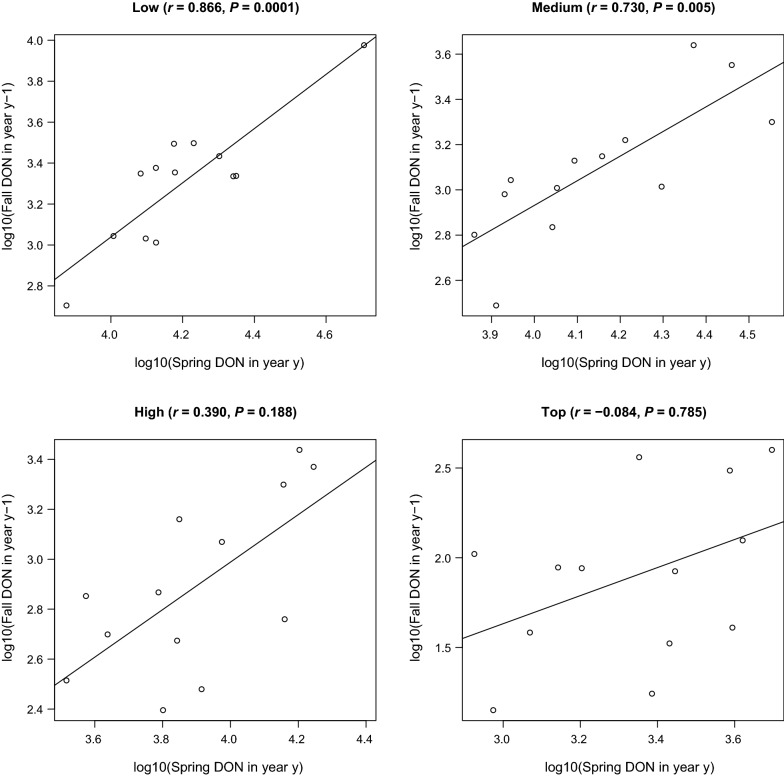
Correlation plots showing the relationship between the fall peak in year *y* − 1 and the spring peak in year *y* for each of the four elevation sites. The fall peak in year *y* − 1 is strongly correlated with the spring peak in year *y* for the low and medium elevation sites. The Pearson correlation coefficient (*r*) and the *p*-value (*P*) are shown in brackets at the top of each panel. These results support the direct development hypothesis and indicate that the questing activity of nymphs belonging to the same cohort (i.e., recruited from larvae that obtained their blood meal in year *y* − 1) starts in the fall of year *y* − 1 and ends in the summer of year *y*, and it does not correspond to the calendar year

Under the developmental diapause hypothesis, we predict that the fall peak in year *y* is correlated with the spring peak in year *y*. In contrast, under the direct development hypothesis, we predict that the fall peak in year *y* − 1 is correlated with the spring peak in year *y*. To compare these two competing hypotheses, we created scatter plots of the fall peak versus the spring peak with different time lags and calculated the Pearson correlation coefficient. The fall peak in year *y* − 1 was strongly correlated with the spring peak in year *y* for the low elevation site (Fig. 5; *r* = 0.866, *P* = 0.0001) and the medium elevation site (Fig. 5; *r* = 0.730, *p* = 0.005). In contrast, the fall peak and the spring peak in the same calendar year were not correlated (Additional file 1: Section S4). These results support the direct development hypothesis and provide strong evidence that the fall peak and spring peak that bookend the same winter represent the same generation of ticks.

### GAMs with a site-specific smoother of calendar day model the bimodal phenology of the DON

 We found that the bimodal phenology of the DON was best captured using GAMs that contained a site-specific smoother function of the calendar day rather than temperature, relative humidity, or saturation deficit. Visual inspection shows that the values predicted by this site-specific smoother function of calendar day re-created the bimodal or unimodal phenology of the DON at each of the four elevation sites (Additional file 1: Section S5).

### Description of the set of base models

For the first step, we compared 33 base models that contained the fixed factors elevation site, nymphal peak, beech masting index, and year. All 33 models contained a site-specific smoother function of the calendar day (1 January and 31 December corresponded to day 1 and day 365). The 33 models differed with respect to the beech masting index (BM[2,2], BM[1,1], and BM[2,1]), the year:site interaction, and the two-way and three-way interactions between elevation site, nymphal peak, and the beech masting index.

### Description of the best base model

The model selection table for the top 12 base models is presented in Table 5 (the complete model selection table for the 33 base models is shown in Additional file 1: Section S6). The best base model had the second lowest AICc value (4483.632), a support of 22.95%, and an adjusted *r*^2^ value of 68.52%, and it explained 76.7% of the deviance (model 2 in Table 5). This model contained the fixed effects of elevation site, nymphal peak, BM[2,1], the covariate year, and the interaction between elevation site and year (model 2 in Table 5). The top four base models accounted for 92.03% of the support, and they only differed with respect to their two-way interaction involving BM[2,1] (Table 5). For example, models 1, 3, and 4 contained model 2 and the interaction between nymphal peak and BM[2,1], the interaction between elevation site and BM[2,1], and both these two-way interactions, respectively (Table 5).

**Table 5:** Model selection results are shown for the generalized additive model (GAM) with negative binomial errors of the density of *I. ricinus* nymphs (DON) at the four elevation sites on Chaumont Mountain over 14 years (2004 to 2017)

Rank	Model structure	*df*	logLik	AICc	ΔAICc	Weight1 (%)	Weight2 (%)	*r*^2^ (%)
1	S + P + B_2.1 + Y + S:Y + P:B_2.1	40	−2197.872	4483.411	0.000	25.63	25.63	68.78
2	S + P + B_2.1 + Y + S:Y	39	−2199.014	4483.632	0.221	22.95	48.58	68.52
3	S + P + B_2.1 + Y + S:Y + S:B_2.1	42	−2195.501	4483.645	0.234	22.80	71.39	68.53
4	S + P + B_2.1 + Y + S:B_2.1 + P:B_2.1 + S:Y	43	−2194.543	4483.844	0.433	20.64	92.03	68.93
5	S + P + B_2.1 + Y + S:P + P:B_2.1 + S:Y	43	−2196.813	4488.264	4.853	2.26	94.29	68.64
6	S + P + B_2.1 + Y + S:P + S:B_2.1 + S:Y	45	−2194.473	4488.622	5.211	1.89	96.19	68.39
7	S + P + B_2.1 + Y + S:Y + S:P	42	−2198.053	4488.631	5.220	1.88	98.07	68.39
8	S + P + B_2.1 + Y + S:P + S:B_2.1 + P:B_2.1 + S:Y	46	−2193.434	4488.697	5.286	1.82	99.89	68.78
9	S × P × B_2.1 + Y + S:Y	49	−2192.736	4494.377	10.966	0.11	100.00	68.53
10	S + P + B_1.1 + Y + S:Y + P:B_1.1	39	−2221.763	4529.831	46.419	0.00	100.00	63.90
11	S + P + B_1.1 + Y + S:B_1.1 + P:B_1.1 + S:Y	42	−2220.213	4533.738	50.327	0.00	100.00	63.97
12	S + P + B_1.1 + Y + S:P + P:B_1.1 + S:Y	42	−2220.971	4535.077	51.666	0.00	100.00	63.71

The explanatory variables were elevation site (S), nymphal peak (P), beech masting index (B), and year (Y). There are three different beech masting indices, B_2.2, B_1.1, and B_2.1, which reflect the different time lags for the spring peak and fall peak. Interactions are indicated with a full colon; for example, the interaction between site and year is represented with ‘S:Y’. To model the bimodal phenology of the DON, a site-specific smoother function was applied to the calendar day, s(day, by = S); this site-specific smoother function is common to all the models and is therefore not shown in the model structure. The models are ranked according to their corrected Akaike information criterion (AICc). Shown for each model are the model rank (Rank), model structure, model degrees of freedom (*df*), log-likelihood (logLik), AICc, difference in the AICc value from the top model (ΔAICc), model weight (Weight1), cumulative model weight (Weight2), and adjusted *r*-squared value (*r*^2^)

### Comparison of the direct development hypothesis and the developmental diapause hypothesis

**Fig. 6:**
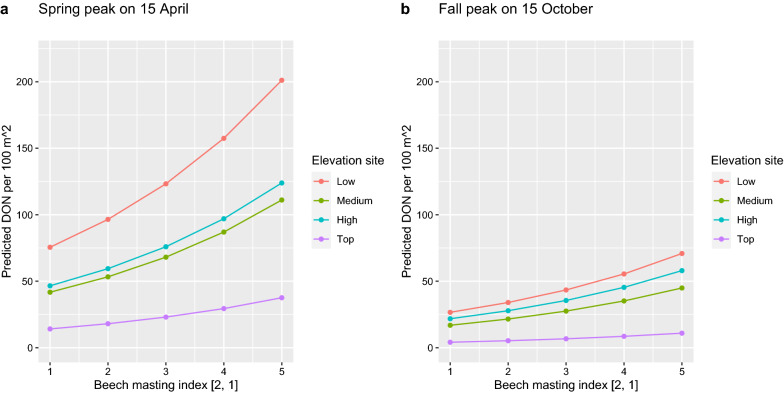
Effect of beech mast index with different time lags (2 years versus 1 year) for the spring and fall peak (BM[2,1]) on the density of nymphs (DON) is shown for the (**a**) spring peak and (**b**) fall peak. The beech mast index (BM[2,1]) assumes a 2-year time lag for the spring nymphal peak and a 1-year time lag for the fall nymphal peak. The relationship shows that the DON increases with the beech mast index at each of the four elevation sites for both the (**a**) spring peak and (**b**) fall peak. The DON is an estimate of the number of questing *I. ricinus* nymphs per 100 m^2^ sampled by the dragging method each month. Beech mast indices have values of 1, 2, 3, 4, and 5, which refer to very poor mast, poor mast, moderate mast, good mast, and full mast, respectively. The predicted values were calculated using model 2 in Table 5. The reference conditions were as follows: year = 2004, and calendar days were 105 (15 April) and 288 (15 October) for the spring and fall peak, respectively. Increasing the beech mast score from 1 (poor mast) to 5 (full mast) increased the DON by 166% at each of the four elevation sites

We found very strong support for the BM[2,1] index and no support for the other two beech masting indices (BM[2,2] and BM[1,1]). The top nine models had 100% of the support and all included the BM[2,1] index (Table 5). As expected, the BM[2,1] index had a positive and highly significant effect on the DON (Table 6: slope = 0.245, SE = 0.018, *P* < 2e−16). We graphed the predicted values of the DON versus the BM[2,1] index for each of the four elevation sites in both the spring and fall (Fig. 6). For the spring and fall, the reference conditions were that year = 2004, and that the DON was calculated for the midpoint of the spring (15 April) and fall (15 October), respectively. Increasing the BM[2,1] index from 1 (poor mast) to 5 (full mast) increased the DON by 166% at each of the four elevation sites on Chaumont Mountain (Fig. 6). In summary, the inter-annual variation in the spring and fall nymphal peaks in the same calendar year is best explained by masting events that occurred 2 years prior and 1 year prior, respectively. This result suggests that the spring and fall nymphs in calendar year *y* obtained their larval blood meal in year *y* − 1 and year *y*, respectively, and are therefore most likely from different cohorts (i.e., hatched as larvae in different calendar years). This result provides strong evidence for the direct development hypothesis of the origin of the fall peak of nymphs.

**Table 6:** The parameter estimates from the best model (model 2 in Table 5) are shown

Type	Name	Estimate	SE	*z*	*P*
Intercept	Low site	2.477	0.160	15.459	< 2e−16
Contrast 1	Medium–Low	−0.260	0.175	−1.486	0.137
Contrast 2	High–Low	−0.076	0.177	−0.429	0.668
Contrast 3	Top–Low	−1.064	0.192	−5.529	< 0.001
Contrast 4	Fall–Spring	0.693	0.264	2.629	0.009
Slope 1	BM[2,1]	0.245	0.018	13.288	< 2e−16
Slope 2	Year (for Low site)	0.057	0.014	3.999	< 0.001
Contrast 5	Medium site: Year	−0.006	0.020	−0.317	0.751
Contrast 6	High site: Year	−0.082	0.020	−4.016	< 0.001
Contrast 7	Top site: Year	−0.162	0.022	−7.226	< 0.001

Smooth terms		*Edf*	*Ref. df*	*F*	*P*
s(Day)—Low site		7.734	8.590	297.400	< 2e−16
s(Day)—Medium site		6.786	7.904	347.900	< 2e−16
s(Day)—High site		6.823	7.918	237.900	< 2e−16
s(Day)—Top site		5.909	7.043	182.300	< 2e−16

In this best model, the DON response variable was modelled as a function of elevation site (low, medium, high, and top), nymphal peak (spring and fall), covariate beech mast index with different time lags for the spring peak and fall peak (BM[2,1]), and covariate year (rescaled as 1, 2, …, 14). Interactions are indicated by colons (:); for example, the parameter estimates for the site:year interaction are indicated by the terms Medium site: Year, High site: Year, and Top site: Year. For each parameter, the parameter type, parameter name, parameter estimate on the log scale, standard error on the log scale (SE), *z* value (*z*), and *P*-values (*P*) are shown. To model the bimodal phenology of the DON, a site-specific smoother function was applied to the calendar day. For each of the four site-specific smoother functions of the calendar day, the effective degrees of freedom (*Edf*), reference degrees of freedom (*Ref. df*), *F*-statistic (*F*), and *P*-value (*P*) are shown

### Effects of other explanatory variables in the base model

**Fig. 7:**
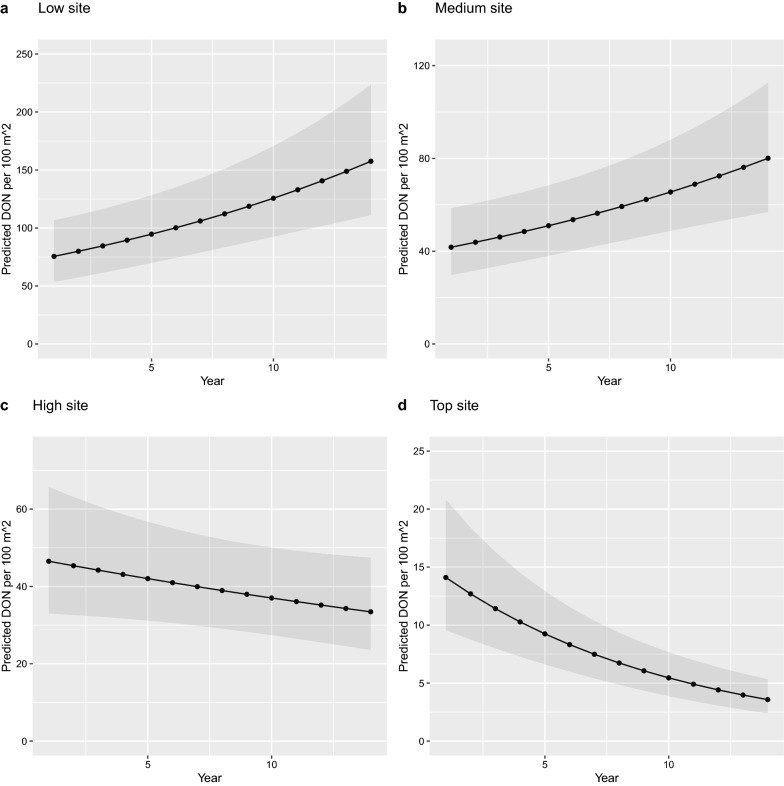
Effect of year on the density of nymphs (DON) is shown for the four elevation sites: (**a**) low site, (**b**) medium site, (**c**) high site, and (**d**) top site. The DON is an estimate of the number of questing *I. ricinus* nymphs per 100 m^2^ sampled by the dragging method each month. Years 1 and 14 refer to years 2004 and 2017, respectively. The predicted values were calculated using model 2 in Table 5. The reference conditions were as follows: season = Spring, BM[2,1]  = 1, calendar day = 15 April. Over the 14-year study period, the DON increased by 108.6% at the low elevation, increased by 92.0% at the medium elevation, decreased by 28.1% at the high elevation, and decreased by 74.7% at the top elevation

For the best base model (model 2 in Table 5), elevation site (Δ*df* = 3, Δdev = 35.656, *P* < 1e−07), year (Δ*df* = 1, Δdev = 15.989, *P* < 0.0001), and their interaction (Δ*df* = 3, Δdev = 67.567, *P* < 1e−13) all had significant effects on the monthly DON. The interaction between elevation site and year indicated that the change in the DON over time differed among the four elevation sites (Fig. 7; Table 6). Over the 14-year period (2004–2017), the DON increased at the low elevation (Table 6: slope = 0.057 per year, SE = 0.014, *P* < 0.001) and medium elevation (Table 6: Medium–Low contrast of the slope =  −0.006, SE = 0.020, *P* = 0.751) sites, but decreased at the high elevation (Table 6: High–Low contrast of the slope =  −0.082, SE = 0.020, *P* < 0.001) and top elevation (Table 6: Top–Low contrast of the slope =  −0.162, SE = 0.022, *P* < 0.001) sites. Over the 14-year period (2004–2017), the DON increased by 108.6% and 92.0% at the low and medium elevation sites but decreased by 28.1% and 74.7% at the high and top elevation sites, respectively (Fig. 7). The difference in intercepts between the fall and spring was positive (Table 6: Fall–Spring contrast = 0.693, SE = 0.264, *P* = 0.009). This counter-intuitive result is caused by the redundancy between the fixed effects structure and the smoother function and is explained in Additional file 1: Section S7.

### Identification of climate variables

To identify two climate variables that influence the monthly DON, we used two sequential rounds of model selection (see Additional file 1: Section S8). We performed only two rounds of model selection to limit model complexity. In the first round, we created a first set of models that consisted of the best base model and a smoother function of one of the 66 climate variables. In the second round, we updated the base model by adding the best climate variable identified in the first round. We then created a second set of models that consisted of the updated base model and a smoother function of one of the remaining 65 climate variables. For each round of model selection, we used three types of smoother functions: no interaction, separated by nymphal peak, or separated by elevation site.

The best climate model had a nymphal peak-specific smoother for the field-collected temperature at the time of tick sampling (*t*) and a site-specific smoother for the SN_Y1_, which is the total snowpack accumulated during the previous year (i.e., a 1-year time lag with respect to the DON). SN_Y1_ is calculated from 1 October to 31 May, and in Fig. 3, SN_Y1_ corresponds to interval *T*_F2_ + *T*_W1_ + *T*_L1_ (excluding the month of September from *T*_F2_). This model had an AICc value of 4319.6 (164.0 units lower than the original base model), a support of 75.0%, an adjusted *r*^2^ value of 73.8%, and explained 84.2% of the deviance (see Additional file 1: Section S8). A comparison of the observed values versus the predicted values of the DON over the 14-year study period for each of the four elevation sites is shown in Additional file 1: Section S9.

**Fig. 8:**
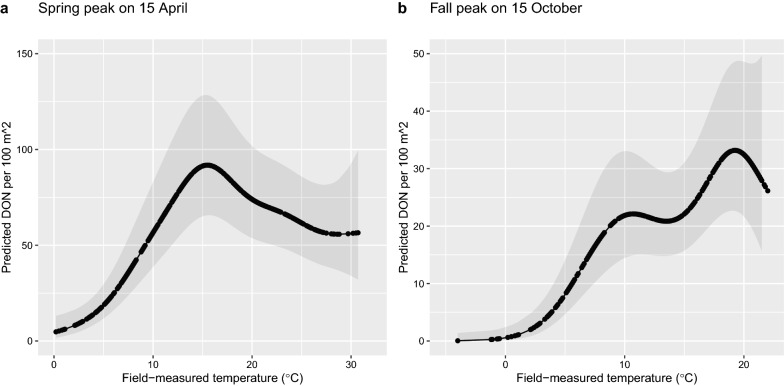
Effect of the field-measured temperature on the density of nymphs (DON) is shown for the (**a**) spring peak and (**b**) fall peak. The smoother function shows that the DON increases with the field-measured temperature and reaches a maximum at ~ 15 °C and ~ 18 °C for the spring and fall nymphal peaks, respectively. The DON is an estimate of the number of questing *I. ricinus* nymphs per 100 m^2^ sampled by the dragging method each month. The field-measured temperature has units of °C and was measured at 60 cm above the ground at the field site on the day of tick sampling. The DON is predicted for the range of field-measured temperatures that occur in the spring and fall. The predicted values were calculated using model 1 in Additional file 1: Table S5. The reference conditions were as follows: site = Low, BM[2,1]  = 1, year = 2004, SN_Y1_ = 150 cm, and calendar days for the Spring and Fall were 15 April and 15 October, respectively

**Fig. 9:**
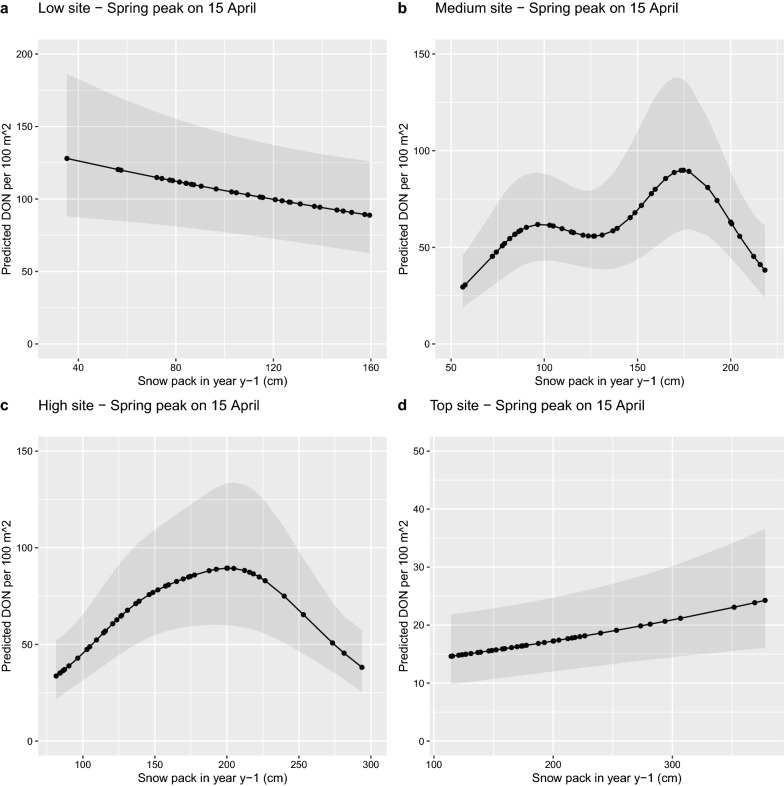
Effect of the total snowpack in year *y* − 1 (SN_Y1_) on the density of nymphs (DON) is shown for the four elevation sites: (**a**) low site, (**b**) medium site, (**c**) high site, and (**d**) top site. The DON is an estimate of the number of questing *I. ricinus* nymphs per 100 m^2^ sampled by the dragging method each month. The SN_Y1_ refers to the total snowpack in the previous year as the DON (i.e., a time lag of 1 year). The SN_Y1_ has units of cm and was measured at 200 cm above the ground by two weather stations near the field site. The DON is predicted for the range of SN_Y1_ values that occur at each elevation site. The effect of SN_Y1_ on the DON differs among the four elevation sites. The predicted values were calculated using model 1 in Additional file 1: Table S5. The reference conditions were as follows: season = Spring, BM[2,1]  = 1, year = 2004, field-measured temperature = 15 °C, and calendar day was 15 April

For the field-collected temperature and SN_Y1_ we graphed the predicted values of the DON over the relevant range of the climate variables for the nymphal peak (Fig. 8) and the four elevation sites (Fig. 9), respectively. Figure 8 shows that the DON increased with the temperature on the day of tick sampling and reached a peak at ~ 17 °C in both the spring peak and the fall peak. Figure 9 shows that the relationship between the DON and SN_Y1_ differed among the four elevation sites. The relationship between SN_Y1_ and the DON was decreasing for the low elevation site, hump-shaped for the medium and high elevation sites, and increasing for the top elevation site (Fig. 9).

### Summary of the results

In summary, the DON had a bimodal phenology at the low, medium, and high elevation sites and a unimodal phenology at the top elevation site. The DON increased over the 14 years of the study at the low and medium elevation sites, whereas it decreased over time at the high and top elevation sites. The DON increased significantly with the beech masting index, and the time lag differed between the two nymphal peaks with a 2-year time lag for the spring peak and a 1-year time lag for the fall peak. Thus, the spring and fall nymphs in calendar year *y* obtained their larval blood meal in year *y* − 1 and year *y*, respectively, which suggests that they are from different generations. While we have no direct evidence, the most parsimonious explanation is that the spring and fall nymphs in calendar year *y* came from larvae that hatched in year *y* − 1 and year *y*, respectively (i.e., in the same year that they obtained their blood meal). This result provides strong evidence for the direct development hypothesis and it contradicts the developmental diapause hypothesis. The DON increased with the field-measured temperature but reached a plateau at ~ 17 °C. Finally, the relationship between the DON and the total snowpack accumulated during the previous year (SN_Y1_) was complex, and it differed among the four elevation sites.

### Fall nymphs have higher fat content than spring nymphs

**Fig. 10:**
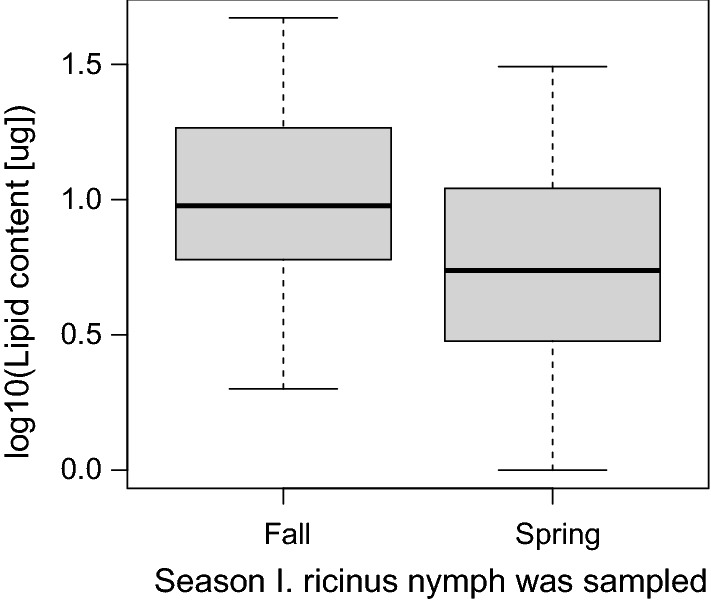
Questing *Ixodes ricinus* nymphs collected in the fall have a higher fat content compared to nymphs collected in the spring of the same calendar year. This result suggests that the fall questing nymphs are younger than the spring questing nymphs. The boxplot shows the medians (black line), the 25th and 75th percentiles (edges of the box), and the minimum and maximum values (whiskers)

We compared the log10-transformed fat content between the *I. ricinus* nymphs collected in the fall of 2010 and the nymphs collected in the spring of 2010 using a two-independent-samples *t*-test. The mean fat content of the fall nymphs (*n* = 40; mean = 9.3 µg; 95% CI = 7.0 to 12.4) was 76.1% higher compared to the spring nymphs (*n* = 40; mean = 5.3 µg; 95% CI = 4.0 to 7.1), and this difference was significant (Fig. 10; *t* = 2.653, *df* = 68.003, *P* = 0.010). This result suggests that the fall nymphs are younger than the spring nymphs, which is consistent with the direct development hypothesis.

## Discussion

Given the importance of *I. ricinus* as a disease vector, forecasting the density of ticks questing for hosts is important for managing the risk of tick-borne diseases [15, 16, 38, 39, 80–82]. In Europe, there is much interest in determining which ecological factors influence the seasonal and inter-annual abundance of *I. ricinus* ticks [15, 38]. In continental Europe, *I. ricinus* nymphs often have a bimodal phenology, with a large spring peak followed by a smaller fall peak. There are at least two alternative hypotheses to explain the fall peak of nymphs: developmental diapause and direct development (Fig. 1). We provide multiple lines of evidence that the fall peak is best explained by the direct development hypothesis at our field site in Switzerland. The fall peak of nymphs does not occur at our top elevation site (Fig. 4), and we speculate that summer temperatures at the top site are not sufficiently warm to permit direct development of engorged larvae into nymphs. The fall peak in year *y* − 1 is strongly correlated with the spring peak in year *y*, indicating that they represent the same tick generation (Fig. 5). Inter-annual variation in the fall peak is best explained by a 1-year time lag with beech masting compared to the standard 2-year time lag for the spring peak (Fig. 6). Fall nymphs have higher fat content than spring nymphs, indicating that they are younger (Fig. 10). All these results are consistent with the direct development hypothesis, and they are inconsistent with the developmental diapause hypothesis, as we explain in more detail below.

### *Ixodes ricinus* nymphs at Chaumont Mountain have a bimodal phenology

The seasonal phenology of *I. ricinus* nymphs at Chaumont Mountain is bimodal, where a large spring peak is followed by a smaller fall peak. This bimodal phenology occurred at the three lower elevation sites, whereas a unimodal phenology with a single large peak in the spring occurred at the top elevation site (Fig. 4), and this pattern is consistent with the direct development hypothesis. The mean field-measured temperature in the spring and summer at the top elevation site is 5.4 °C colder compared to the low elevation site. As a result, tick development rates at the top site are slower, engorged larvae rarely (if ever) undergo direct development to questing nymphs in the same calendar year, and there is no fall peak of nymphs. Across Europe, we expect *I. ricinus* populations to have a bimodal phenology, where longer, warmer summers facilitate direct development of engorged larvae into unfed nymphs in the summer so that they have enough time to quest and create the fall nymphal peak that same year (Table 1). In contrast, we expect a unimodal phenology to occur at northern latitudes or at high elevation sites (Table 1), where lower temperatures during the summer do not allow engorged larvae to complete their development from engorged larvae to unfed nymphs and quest in the same calendar year [43, 53, 83].

Our study also found that *I. ricinus* nymphs started questing earlier at the low elevation site (April) compared to the medium, high, and top elevation sites (May), and we expect this to also be true for the larvae. Thus, larvae at the low site start questing earlier, which means that they have more time to complete their larva-to-nymph moult and they have faster development rates due to the elevated summer temperatures compared to larvae at the top elevation site. All these differences combine to create a fall nymphal peak that is prominent at the low elevation site and absent at the top elevation site, and this altitudinal gradient in phenology is consistent with the direct development hypothesis.

### One cohort of larvae quests as nymphs in two different calendar years

A striking result of our study is the strong correlation between the fall nymphal peak in year *y* − 1 and the spring nymphal peak in year *y* at both the low and medium elevation sites (Fig. 5). In contrast, there is no correlation between the spring peak and the fall peak in the same calendar year (Additional file 1: Section S4). These results are consistent with the direct development hypothesis, and they are not consistent with the developmental diapause hypothesis (Fig. 1). Under the direct development hypothesis, the larvae that obtain an early larval blood meal and/or that develop rapidly in year *y* − 1 will quest as nymphs in the fall of year *y* − 1, whereas the larvae that obtain a late larval blood meal and/or that develop slowly in year *y* − 1 will overwinter as unfed nymphs and quest in the spring of year *y*. Under the direct development hypothesis, the questing activity of nymphs belonging to the same cohort (i.e., recruited from larvae that obtained their blood meal in year *y* − 1) starts in the fall of year *y* − 1 and ends in the summer of year *y*, and it does not correspond to the calendar year. Studies that analyse inter-annual variation in the density of *I. ricinus* nymphs typically calculate a cumulative DON for the calendar year [15, 38, 39, 47, 49]. The present study shows that this approach is wrong when the fall peak and the spring peak in the same calendar year are two different cohorts of ticks. Researchers studying *I. ricinus* populations with a bimodal phenology should be aware that the spring and fall peaks in the same calendar year could represent two different cohorts of nymphs.

### Beech masting has different time lags with the spring and fall nymphs

An important result is the strong and positive association between the beech masting index (BM_[2,1]_) and the density of *I. ricinus* nymphs in the spring and fall (Fig. 6). For the spring nymphal peak, the consensus is that it consists of nymphs that obtained their larval blood meal the previous spring and summer, moulted into nymphs in the summer and fall [84], entered behavioural diapause, overwintered as unfed nymphs, and quested the following spring [21, 53, 60]. We expect a 2-year time lag between beech masting and the spring nymphal peak, because masting in the fall of year *y* − 2 increases the density of rodents and larval feeding success in the spring and summer of year *y* − 1, which in turn increases the DON in the spring of year *y*. Studies in North America and Europe have shown that the masting events of deciduous trees can drive inter-annual variation in the DON and DIN of *Ixodes* nymphs with a 2-year time lag [17, 35, 36, 38]. We previously showed, using the same data from the present study, that the beech masting index 2 years prior was highly significantly associated with inter-annual variation in the DON and DIN [15, 39]. In these studies, we had incorrectly assumed that the spring and fall peaks that occur in the same calendar year represent the same cohort of nymphs [15, 39]. Our previous studies found a strong association between beech masting 2 years prior and the annual DON because the spring peak is typically six to seven times larger than the fall peak [15, 39]. Nevertheless, our decision to combine the spring and fall peaks of the same calendar year into an estimate of the annual DON and the annual DIN was incorrect [15, 39].

The novel aspect of the present study is that our analysis of the bimodal phenology of the DON shows that the spring peak and the fall peak of the same calendar year represent different cohorts of nymphs. Inter-annual variation in the spring peak and fall peak was best explained by the beech masting index 2 years prior and 1 year prior, respectively. This result provides strong evidence for the direct development hypothesis [41, 45, 46, 53, 60], which predicts a 1-year time lag between beech masting and the fall nymphal peak; masting in year *y* − 1 increases the density of rodents and larval feeding success in year *y*, which in turn increases the DON in the fall of year *y*. In contrast, the developmental diapause hypothesis [21, 53, 60] predicts a 2-year time lag between beech masting and the fall nymphal peak; masting in year *y* − 1 increases the density of mice and larval feeding success in year *y*, but larvae that obtain their blood meal in late summer have to overwinter as engorged larvae and moult into unfed nymphs in the summer of year *y* + 1, which increases the DON in the fall of year *y* + 1. A seminal review on diapause in *Ixodes* ticks presented both hypotheses, but appeared to favour the developmental diapause hypothesis over the direct development hypothesis [21]. In contrast, our AIC-based model selection found that the direct development hypothesis for the fall peak of *I. ricinus* nymphs had 100% support, whereas the developmental diapause hypothesis had 0% support.

### Differences in fat content between spring and fall nymphs support the direct development hypothesis

The direct development hypothesis predicts that the fall nymphs are younger compared to the spring nymphs (~ 3 months versus ~ 9 months since the larval blood meal), which agrees with studies comparing the fat content between these two types of nymphs in our study area and elsewhere [53, 73]. In the present reanalysis of the fat content of field-collected *I. ricinus* nymphs at our study location (Fig. 10), we found that the fall nymphs had 76% more fat content than the spring nymphs [73], and studies in the United Kingdom have found a similar pattern [53]. This phenomenon can be explained by the direct development hypothesis; fall nymphs obtained their larval blood meal earlier that summer and their fat content is high because they are young (~ 3 months since the larval blood meal), with less time to burn their fat reserves. In contrast, spring nymphs obtained their larval blood meal the previous summer and their fat content is low because they are older (~ 9 months since the larval blood meal), with more time to burn their fat reserves. Thus, the finding that fall nymphs have higher fat reserves than spring nymphs is consistent with our discovery of 1-year and 2-year time lags for the fall and spring peak, and both these results support the direct development hypothesis.

Some studies have proposed the summer quiescence hypothesis to explain the bimodal phenology of *I. ricinus* nymphs [12, 45]. Under this hypothesis, nymphal questing activity decreases in the late summer because the nymphs are hiding in the leaf litter from unfavourable conditions (high temperatures and low RH) and waiting to resume their questing activity in the fall when conditions are more favourable [12, 45]. The summer quiescence hypothesis makes the same predictions as the developmental diapause hypothesis, namely that the spring and fall peaks of the same calendar year represent the same cohort of ticks and that the fall nymphs are older than the spring nymphs. The summer quiescence hypothesis cannot explain the bimodal phenology of *I. ricinus* nymphs at our study location, for two reasons. First, if the spring and fall peak are from the same cohort, then the fall nymphs are older and should have lower fat content than the spring nymphs, but this was not the case in our study or other studies [53, 73]. Second, if the spring and fall nymphs are from the same cohort, then they should have the same time lag with respect to the beech masting index, but this was not the case in our study. In summary, we found no evidence that the summer quiescence hypothesis can account for the bimodal phenology of *I. ricinus* nymphs at our study location. Nevertheless, summer quiescence of nymphs may be an important strategy for *I. ricinus* populations to avoid hot and dry summers in other locations.

### Effect of weather on the questing behaviour of *I. ricinus* nymphs

Our study found that the field-measured temperature on the day of tick sampling was positively correlated with the density of questing nymphs (Fig. 8), which agrees with other studies across Europe [42, 85–88]. These studies suggest that for any given date, warmer temperatures increase the percentage of nymphs questing for hosts (i.e., increased nymphal activity levels), which in turn increases the number of questing nymphs captured by drag sampling. *Ixodes ricinus* becomes active above a minimum threshold temperature, and questing activity increases with temperature up to some maximum value. While these threshold temperatures are expected to vary among tick populations from different geographical locations, a previous study at our field location suggested that the maximum threshold value was 24 °C [45], whereas the present study found that this threshold was 17 °C. *Ixodes ricinus* is also sensitive to desiccation during questing [20], and they make repeated return trips to the litter layer where they can rehydrate and maintain their water balance [63, 64, 89]. For this reason, ticks prefer to quest under cool and humid conditions (i.e., low SD) [62, 90], and they reduce their questing activity in hot and dry conditions (i.e., high SD) [45, 50, 68, 91, 92]. Our observation that the DON peaked at ~ 17 °C in our study (Fig. 8) suggests that *I. ricinus* nymphs at our study site avoid questing in hot (and presumably dry) conditions.

### Effect of climate variables on the population ecology of *I. ricinus* ticks

Climate variables can influence tick population ecology via their effects on tick life history traits (development, survival, and reproduction). A limitation of our previous studies was that we only investigated annual climate variables (e.g., mean daily temperature was averaged over the entire calendar year) [15, 39]. The present study is much improved because it compares climate variables operating at different temporal scales (i.e., annual versus seasonal). Many steps in the tick life cycle happen over short time scales, suggesting that the critical climate variables operate over seasons rather than years. Researchers have addressed this issue by investigating a wide variety of durations and time lags [16, 49, 93]. For example, a 14-year study in Switzerland averaged the climate variables over different time intervals (1, 5, 10, 17, or 30 days) to determine the critical time period that influences the DON [49]. An 8-year study in Germany used cross-correlation maps to explore month-to-month correlations between the DON and climate variables, as well as time-lagged and interval-averaged correlations by considering a second time lag [16]. A 2-year study in five European countries examined time-lagged and interval-aggregated monthly and yearly means to test the associations between the abundance of questing *I. ricinus* nymphs and climate [93]. Despite this research effort, there is still uncertainty about which climate variables operating over what time scales drive the population ecology of *I. ricinus*.

Our previous work found that mean annual relative humidity and mean annual precipitation were important for explaining the inter-annual variation in the DON and the DIN [15, 39], but the present study found no support for these two climate variables. In the present study, the second most important climate variable (after the field-measured temperature) was the SN_Y1_, which is the total snowpack accumulated during the previous year (i.e., a 1-year time lag with respect to the DON). This variable is calculated from 1 October to 31 May, and in Fig. 3, SN_Y1_ corresponds to interval *T*_F2_ + *T*_W1_ + *T*_L1_ (excluding the month of September from *T*_F2_). In Figs. 1 and 3, the winter with the 1-year time lag separates the adult female ticks in year *y* − 1 and the larvae in year *y*, which suggests that this climate variable is operating on survival of eggs or on the survival of gravid adult female ticks. Studies on *I. ricinus* populations in Ireland have shown that adult female ticks that engorge in the spring of year *y* − 1 will lay eggs that summer, of which a substantial fraction will overwinter and hatch in the spring of year *y* [60]. Alternatively, adult female ticks that engorge in the fall of year *y* − 1 will overwinter in a gravid state and lay the eggs in the spring of year *y* [60]. Note that in both scenarios, the eggs must survive the winter (either in the environment or inside the gravid adult female) and the resultant larvae hatch in the spring/summer of year *y*. Our study found that the relationship between SN_Y1_ and the DON differed among the four elevation sites (Fig. 9). The relationship was decreasing for the low elevation site, hump-shaped for the medium and high elevation sites, and increasing for the top elevation site (Fig. 9). Previous studies have shown that snowpack can have positive effects on tick survival because it increases ground surface temperatures [94], prevents repeated freeze and thaw cycles, and ensures high and stable relative humidity [95–99]. Interestingly, the effect of increasing snowpack was monotonically positive at the top elevation site, where air temperatures are coldest. The negative effects of high snowpack at the low, medium, and high elevation sites might be caused by snowmelt in spring, which could cause recently hatched larvae or recently laid eggs to drown, as previous studies have shown that high water levels can reduce tick survival and inhibit tick activity [20, 100]. However, we acknowledge that these conclusions are highly speculative, and the reasons are not clear why annual snowpack in the previous year has different effects on the DON at the four elevation sites. Nevertheless, our results demonstrate that the effect of climate variables on the DON can vary dramatically among tick populations located in the same general area but at different elevations.

### GAMs can model the bimodal phenology of *I. ricinus* nymphs

The bimodal non-linear phenology of *I. ricinus* nymphs is a challenge for statistical modelling. In our previous work, we avoided this complexity by analysing the total DON and DIN for each year [15, 39], and the same approach was used in a 14-year study in Switzerland that is near our study site [49], in a 10-year study in the Netherlands [47], and in a 13-year study in North America [17]. An 8-year study in Germany used different intercepts for the spring, summer, fall, and winter to deal with the non-linear phenology of *I. ricinus* nymphs [16]. We demonstrated that generalized additive models (GAMs) are a useful approach to model the bimodal phenology of *I. ricinus* nymphs because they allow the user to flexibly model the DON as a non-parametric and non-linear smoother function of the explanatory variables of interest. The site-specific smoother function of the calendar day was an excellent fit to the seasonal phenology of *I. ricinus* nymphs (Additional file 1: Section S5). A disadvantage of non-parametric smoother functions is that they do not provide parameter estimates (by definition), which makes it difficult to make quantitative statements about the effect sizes of the explanatory variables. Another disadvantage was that the site-specific smoother function of calendar day did such a good job at capturing variation in phenology among the four elevation sites that the parameter estimates for some fixed effects (e.g., nymphal peak) became counter-intuitive (Additional file 1: Section S7). In summary, we demonstrated that the bimodal non-linear phenology of *I. ricinus* ticks can be captured by GAMs using a combination of parametric and non-parametric functions of the explanatory variables of interest.

## Conclusions

At our study location, the *I. ricinus* populations have a bimodal phenology at the lower elevation sites, but a unimodal phenology at the top elevation site. At the low and medium elevation sites, the fall nymphal peak in year *y* − 1 was strongly correlated with the spring nymphal peak in year *y* over the 15 years of the study. In contrast, there was no correlation between the fall peak and the spring peak in the same calendar year. Inter-annual variation in the fall nymphal peak and spring nymphal peak were strongly associated with the beech masting index 1 year prior and 2 years prior, respectively. Fall nymphs had higher fat content than spring nymphs, indicating that they are younger in age. All these results support the direct development hypothesis, and they contradict the developmental diapause hypothesis. In areas with a bimodal phenology, the DON and risk of tick-borne disease will increase 1 year later in the fall and 2 years later in the spring following a full mast year. Studies on the population ecology of *I. ricinus* should consider that the nymphal peaks in the fall of year *y* − 1 and the spring of year *y* likely represent the same cohort of nymphs. For *I. ricinus* populations with a bimodal phenology, studies that calculate the annual DON over the calendar year are likely wrong because they unwittingly combine nymphs belonging to different generations.

## Supplementary Information

**Additional file 1: Section S1.** Interpolation of the climate data from the weather stations. **Section S2.** Goodness of fit for the best model. **Section S3.** Effect of elevation site on the density of *I. ricinus* nymphs. **Section S4.** Correlation plots between the fall and spring nymphal peaks with different time lags. **Section S5.** Site-specific smoother function of calendar day predicts the bimodal or unimodal phenology of *I. ricinus* nymphs at the four elevation sites. **Section S6.** AIC-based model selection of the base model. **Section S7.** Interpretation of the parameter estimates of the best model. **Section S8.** AIC-based model selection of climate variables. **Section S9.** Comparison of the observed versus the predicted values of the DON over the 14-year study period for each of the four elevation sites. **Section S10.** Auto-correlation of the residuals.

## Data Availability

The raw data for this study are stored in the Additional file 2. The climate data are available from the Climap-net database of the Federal Office for Meteorology and Climatology (http://www.meteosuisse.admin.ch/home/service-et-publications/conseil-et-service/portail-de-donnees-dedie-aux-specialistes.html). The MASTREE database is available in the Ecology—Ecological Society of America repository (http://onlinelibrary.wiley.com/doi/10.1002/ecy.1785/suppinfo).

## Abbreviations

AIC: Akaike information criterion
ASL: Above sea level
BM: Beech masting
DIN: Density of infected nymphs
DON: Density of nymphs
GAMs: Generalized additive models
PR: Precipitation
RH: Relative humidity
SD: Saturation deficit
SN: Snowfall
T: Temperature
WMO: World Meteorological Organization
