# Supplementary material for: Beech tree masting explains the inter-annual variation in the fall and spring peaks of Ixodes ricinus ticks with different time lags
Source: Parasit Vectors. 2021 Nov 8;14:570. doi: 10.1186/s13071-021-05076-8 (PMC8577035; doi:10.1186/s13071-021-05076-8)
Supplement: Supplementary file 1 — Additional file 1: Section S1. Interpolation of the climate data from the weather stations. Section S2. Goodness of fit for the best model. Section S3. Effect of elevation site on the density of I. ricinus nymphs. Section S4. Correlation plots between the fall and spring nymphal peaks with different time lags. Section S5. Site-specific smoother function of calendar day predicts the bimodal or unimodal phenology of I. ricinus nymphs at the four elevation sites. Section S6. AIC-based model selection of the base model. Section S7. Interpretation of the parameter estimates of the best model. Section S8. AIC-based model selection of climate variables. Section S9. Comparison of the observed versus the predicted values of the DON over the 14-year study period for each of the four elevation sites. Section S10. Auto-correlation of the residuals. [file 13071_2021_5076_MOESM1_ESM.docx]

**Additional File 1**

Manuscript title: Beech tree masting explains the inter-annual variation in the fall and spring peaks of *Ixodes ricinus* ticks with different time lags

Authors: Cindy Bregnard, Olivier Rais, Coralie Herrmann, Olaf Kahl, Katharina Brugger, and Maarten J. Voordouw

**Table of Contents**

[SECTION S1 – Interpolation of the climate data from the weather stations 2](#_Toc84657753)

[SECTION S2 – Goodness of fit for the best model 3](#_Toc84657754)

[SECTION S3 – Effect of elevation site on the density of *I. ricinus* nymphs 4](#_Toc84657755)

[SECTION S4 – Correlation plots between the fall and spring nymphal peaks with different time lags 5](#_Toc84657756)

[SECTION S5 – Site-specific smoother function of calendar day predicts the bimodal or unimodal phenology of *I. ricinus* nymphs at the four elevation sites 8](#_Toc84657757)

[SECTION S6 – AIC-based model selection of the base model 9](#_Toc84657758)

[SECTION S7 – Interpretation of the parameter estimates of the best model 12](#_Toc84657759)

[SECTION S8 – AIC-based model selection of climate variables 13](#_Toc84657760)

[SECTION S9 – Comparison of the observed versus the predicted values of the DON over the 14-year study period for each of the 4 elevation sites 17](#_Toc84657761)

[SECTION S10 – Auto-correlation of the residuals 21](#_Toc84657762)

# SECTION S1 – Interpolation of the climate data from the weather stations

**Methods:** We obtained climate data from the Federal Office of Meteorology and Climatology MeteoSwiss using the CLIMAP-net application. Climate data were obtained from two weather stations that are close to our four elevation sites and that are located at 485 m ASL in Neuchâtel (WMO number = 06604) and at 1136 m ASL in Chaumont (WMO number = 06608). To create a climate profile that was specific for each of the four elevation sites, we interpolated the values between the two weather stations using the relative elevation distance of each elevation site to the two weather stations, as we have done previously (Bregnard et al. 2020, Bregnard et al. 2021). For example, the total elevation distance between the Neuchâtel and Chaumont weather stations is 651 meters, and the elevation distance between the top site and the Neuchâtel weather station is 620 meters, which represents 95.2% of the elevation distance. Thus, the climate at the top site is expected to be more like the Chaumont weather station (95.2%) compared to the Neuchâtel weather station (4.8%), whereas the reverse would be true for the low site. For each elevation site, the mean daily temperature, relative humidity, saturation deficit, precipitation and snowfall were calculated based on the interpolating percentages (**Table S1**).

Table S1. Interpolation of the climate data from the two weather stations. Shown are the site, elevation (in meters), elevation distance with the Neuchâtel weather station (Dist 1, in meters), elevation distance between Neuchâtel and Chaumont weather station (Dist 2, in meters), the interpolating percentage from the Neuchâtel weather station (Neuchâtel), and the interpolating percentage from the Chaumont weather station (Chaumont).

| **Site** | **Elevation (m)** | **Dist 1** | **Dist 2** | **Neuchâtel** | **Chaumont** |
| --- | --- | --- | --- | --- | --- |
| Top | 1073 | 620 | 651 | 4.8% | 95.2% |
| High | 900 | 447 | 651 | 31.3% | 68.7% |
| Medium | 740 | 287 | 651 | 55.9% | 44.1% |
| Low | 620 | 167 | 651 | 74.3% | 25.7% |

# SECTION S2 – Goodness of fit for the best model

**Methods:** We used the gam.check() function in the mgcv package of R to assess the goodness of fit for the best GAM in the model selection table in the main manuscript (model 2 in Table 5 in the main manuscript = model 2 in Table S2).

**Results for nymphal abundance:** For the DON, the residuals of the best GAM (model 2 in Table 5 in the main manuscript = model 2 in Table S2) met the assumptions of the method (Figure S1) and therefore confirmed the goodness of fit.


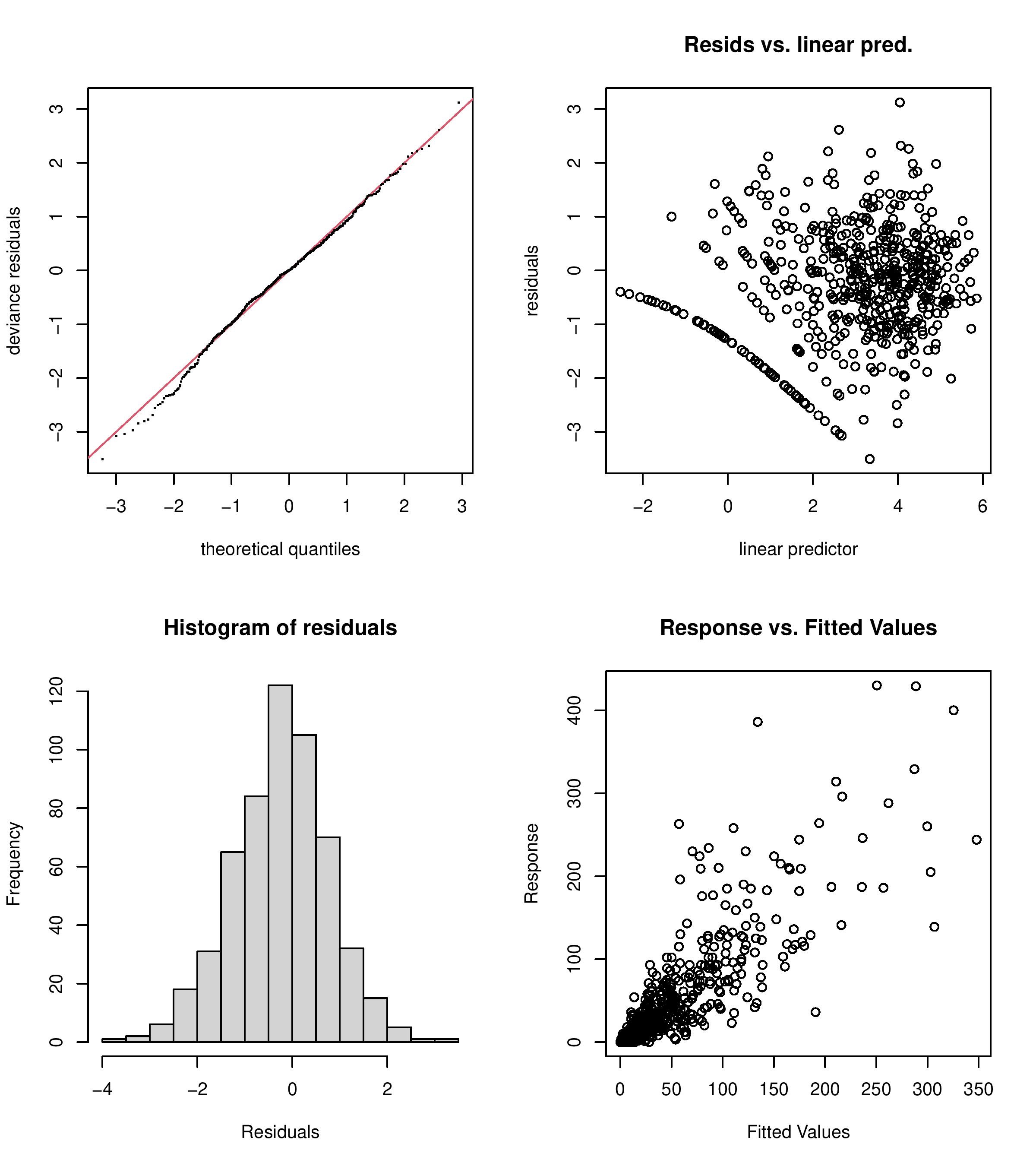


Figure S1. Goodness of fit assumptions for the best GAM in the model selection table of the main manuscript (model 2 in Table 5 in the main manuscript = model 2 in Table S2). The four plots generated by the gam.check() function confirmed that the residuals of the best model generally fit the assumptions of the GAM.

# SECTION S3 – Effect of elevation site on the density of *I. ricinus* nymphs


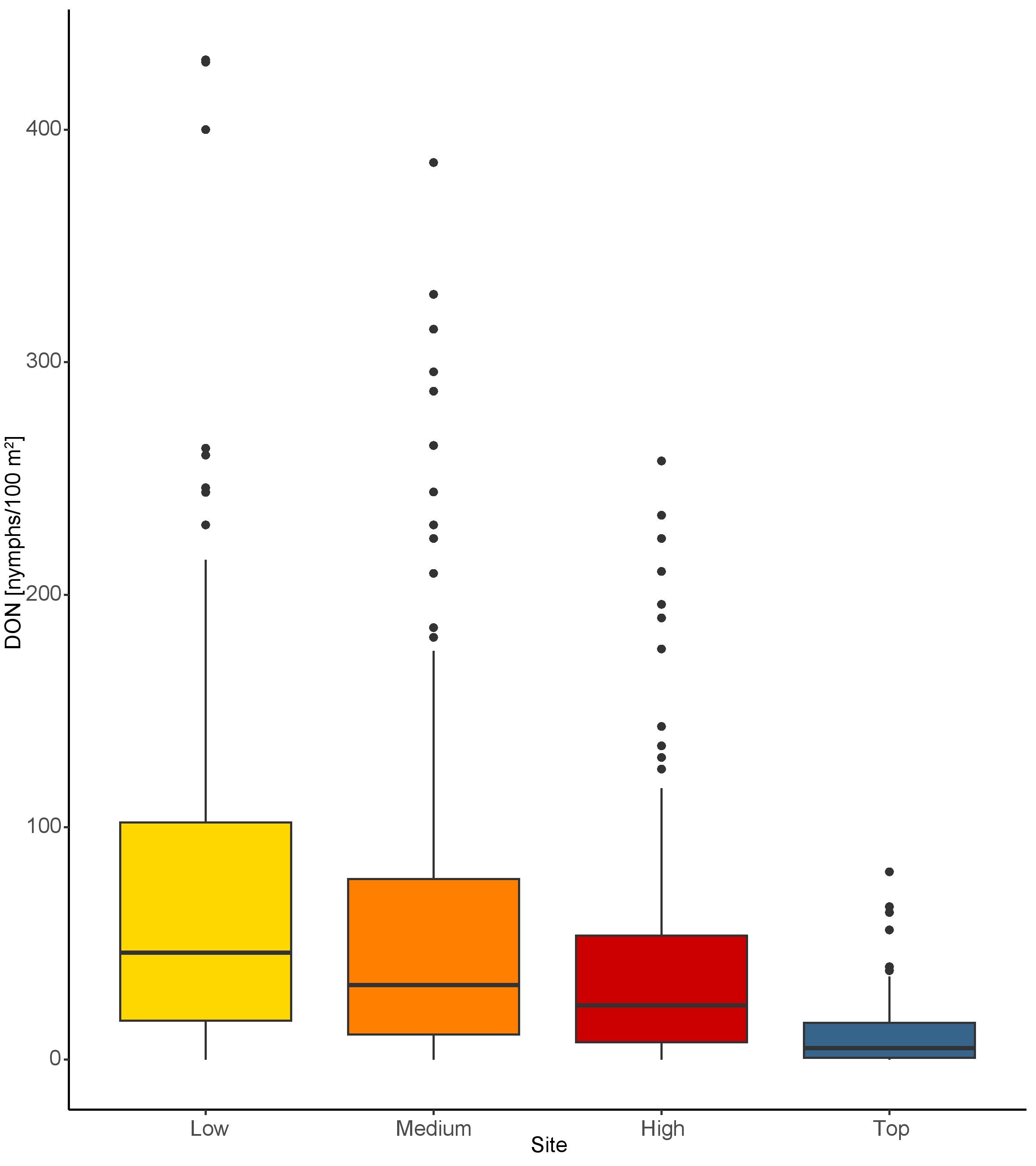
Figure S2. Effect of elevation on the density of nymphs (DON). The DON is an estimate of the number of questing *I. ricinus* nymphs per 100 m^2^ sampled by the dragging method each month. The boxplot shows the medians (black line), the 25th and 75th percentiles (edges of the box), the minimum and maximum values (whiskers), and the outliers (solid circles).

# SECTION S4 – Correlation plots between the fall and spring nymphal peaks with different time lags

**Methods:** Under the direct development hypothesis, the fall peak in year *y*-1 should be correlated with the spring peak in year *y*. In contrast, under the developmental diapause hypothesis, the fall peak in year *y* should be correlated with the spring peak in year *y*. To compare these two competing hypotheses, we created scatter plots of the fall peak versus the spring peak with different time lags and calculated the Pearson correlation coefficient. As an additional control, we tested whether the fall peak in year *y*+1 was correlated with the spring peak in year *y*, even though this correlation pattern is not consistent with any hypothesis.

**Results:** The fall peak in year *y*-1 was strongly correlated with the spring peak in year *y* for the low and medium sites (**Figure 5** in the main manuscript). In contrast, the fall peak and the spring peak in the same calendar year were not correlated (**Figure S3**). Similarly, the fall peak in year *y*+1 was not correlated with the spring peak in year *y* (**Figure S4**). These correlation plots support the direct development hypothesis and they do not support the developmental diapause hypothesis. These correlations show that the questing activity of nymphs belonging to the same cohort (i.e., recruited from larvae that obtained their blood meal in year *y*-1) starts in the fall of year *y*-1 and ends in the summer of year *y* and it does not correspond to the calendar year. Most studies that analyze inter-annual variation in tick abundance calculate the total annual tick abundance over the calendar year. This approach is clearly wrong for our study location.


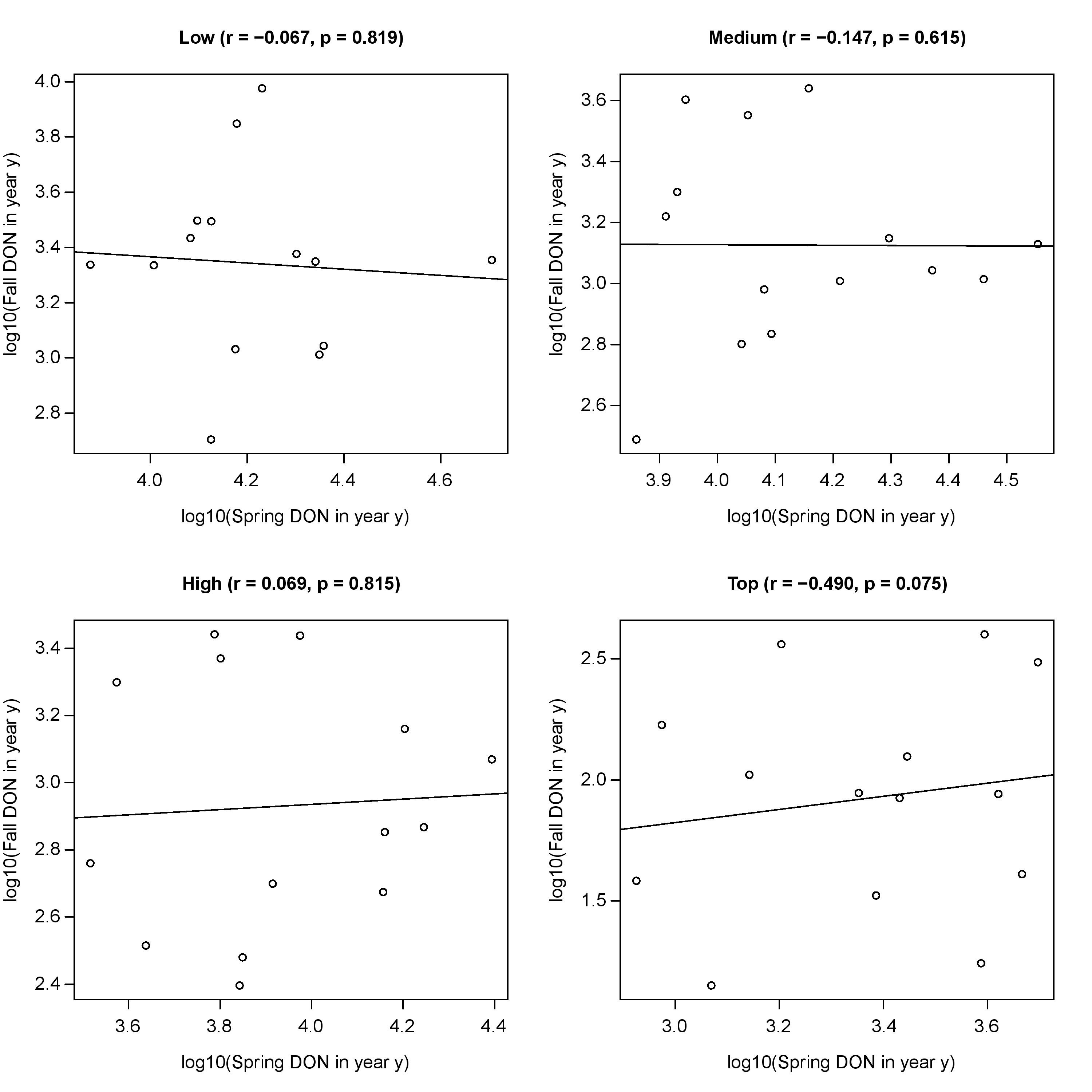


Figure S3. Correlation plot showing the relationship between the fall peak and the spring peak in the same calendar year for each of the four elevation sites. The fall peak in year *y* is not correlated with the spring peak in year *y*. The absence of significant correlations contradicts the developmental diapause hypothesis, which predicts that the fall peak in year *y* will be correlated with the spring peak in year *y*.


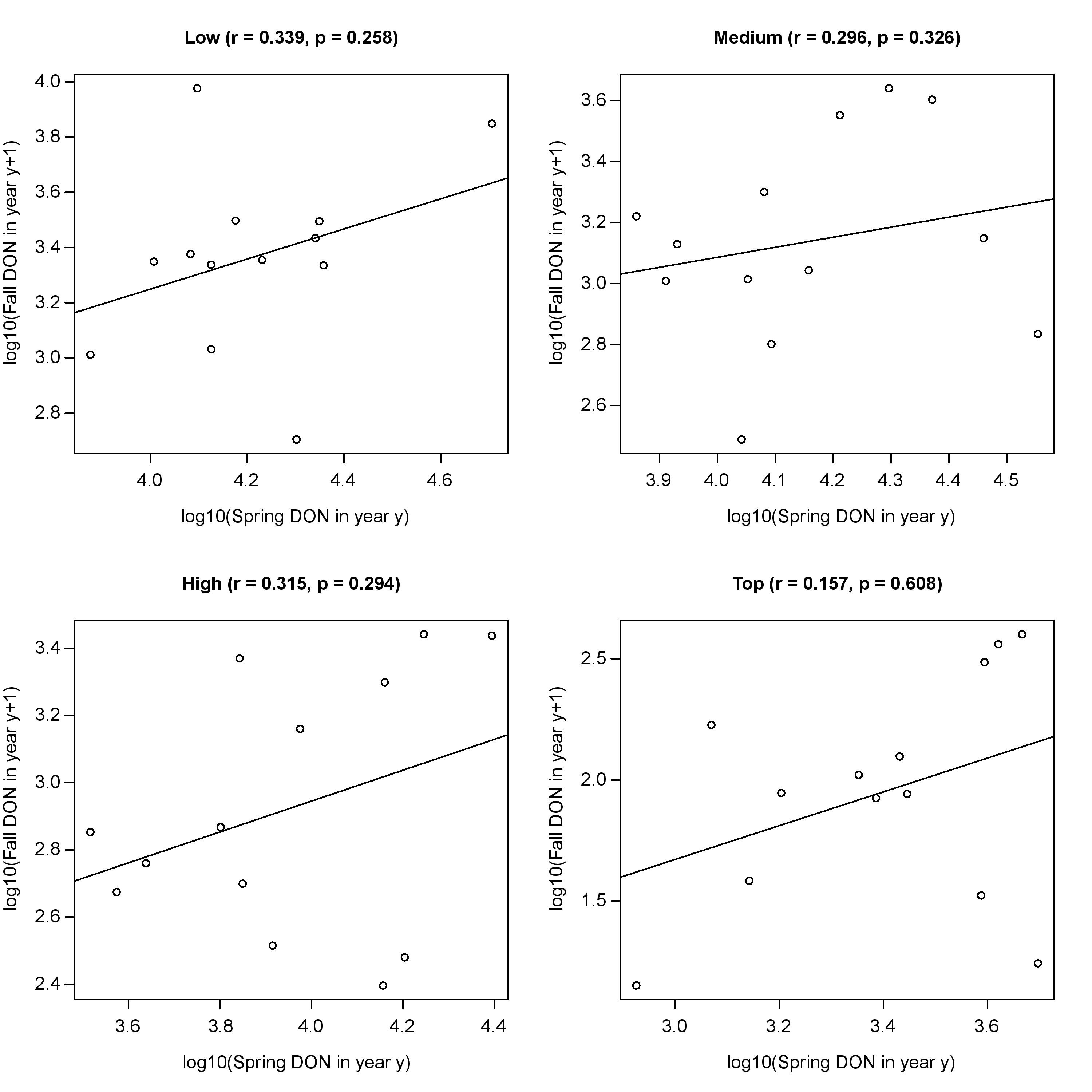
Figure S4. Correlation plot showing the relationship between the fall peak in year *y*+1 and the spring peak in year *y* for each of the four elevation sites. The fall peak in year *y*+1 is not correlated with the spring peak in year *y*. The correlation between the spring peak in year *y* and the fall peak in year *y*+1 is not predicted by any hypothesis about how diapause will influence the phenology of *I. ricinus* nymphs. Figure S4 is a control for Figure S3 and Figure 5 in the main manuscript.

# SECTION S5 – Site-specific smoother function of calendar day predicts the bimodal or unimodal phenology of *I. ricinus* nymphs at the four elevation sites


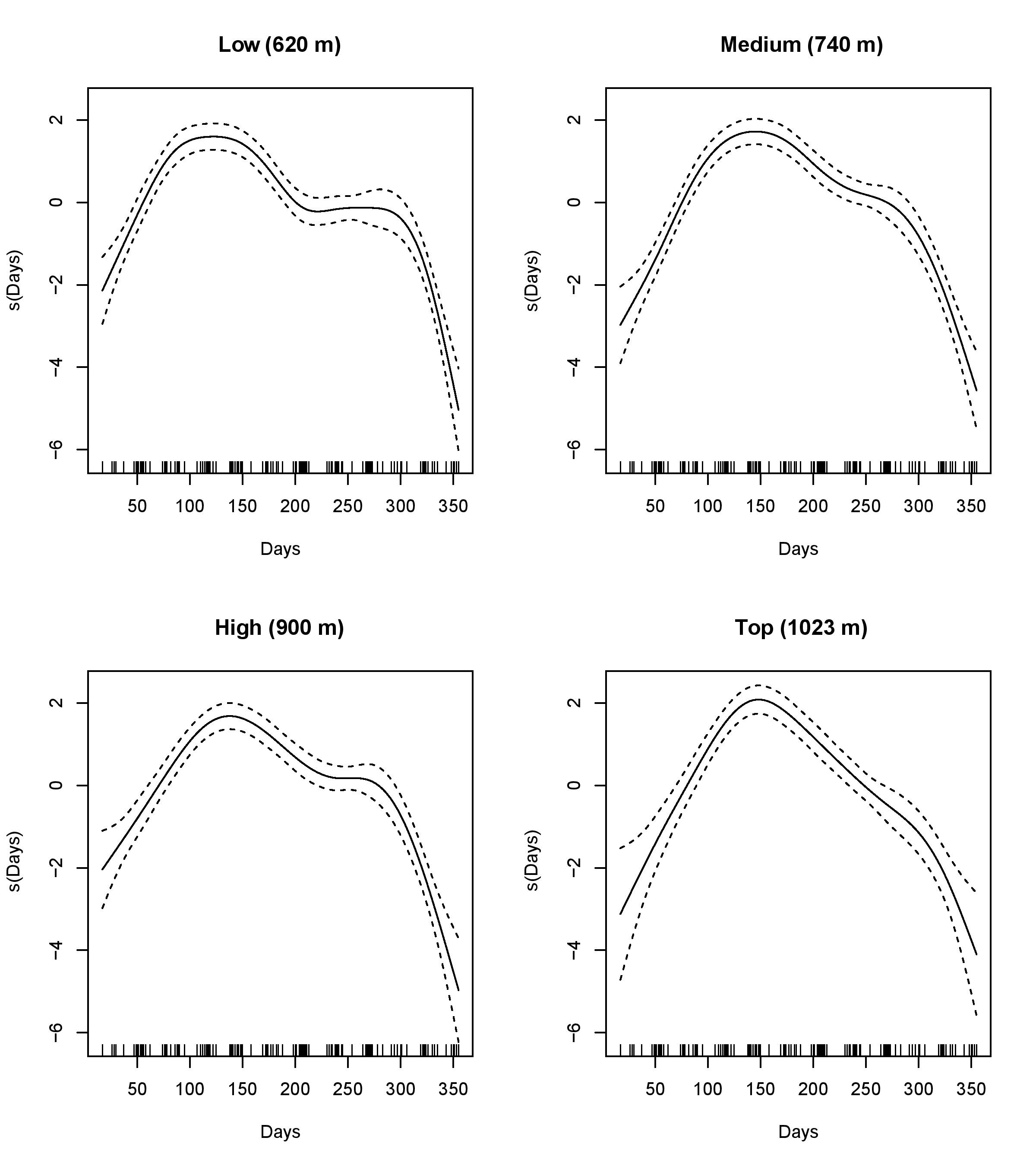
Figure S5. The site-specific smoother of the calendar day is shown for each of the four elevation sites. The smoother function of the calendar day recreates the bimodal phenology for the low, medium, and high elevation sites, whereas the top elevation site has a unimodal phenology.

# SECTION S6 – AIC-based model selection of the base model

To determine the best model for explaining variation in the monthly DON over the 14 years of the study (2004 to 2017) at the four elevation sites on Chaumont Mountain, we used a model selection approach based on the corrected Akaike information criterion (AICc). We used our previous work on the same data set as starting point (Bregnard et al. 2020, Bregnard et al. 2021). This work had calculated the cumulative nymphal density (CND) for each calendar year (i.e., the CND values in **Table 3** in the main manuscript) and found that the inter-annual variation in the CND was best explained by elevation site, year, site:year interaction, and the beech masting score 2 years prior. We therefore included these four explanatory variables in our set of starting models. We added the categorical factor nymphal peak, which had 2 levels: Spring and Fall. We tested 3 different beech masting indices, BM[2,2], BM[1,1], and BM[2,1], where the subscripts [i, j] refer to the time lag between the spring and fall nymphal peaks. The magnitude of the nymphal peak differed between the spring and fall, and the phenology of the DON differed between the 4 elevation sites. For this reason, we included all possible 2-way and 3-way interactions between nymphal peak, elevation site, and beech masting index. We also included the previously mentioned site-specific smoother function of the calendar day to model the bimodal phenology of the DON (**Figure S5**). The original model set contained 33 models that were composed of different combinations of these explanatory variables.

According to AIC-based model selection, the best base model had an AIC score of 4483.632, 22.95% of the support, an adjusted r^2^ value of 68.52%, and it explained 76.7% of the deviance (base model 2 in **Table S2**). The best base model contained the following explanatory variables: elevation site, nymphal peak, BM[2,1], year, site:year interaction, and the site-specific smoothed function of calendar day. In summary, base model 2 confirmed our previous analyses (Bregnard et al. 2020, Bregnard et al. 2021) and that we could model the bimodal phenology of the monthly DON using a site-specific smoother function of calendar day.

Table S2. Model selection results are shown for the generalized additive model (GAM) with negative binomial errors of the density of *I. ricinus* nymphs (DON) at the four elevation sites on Chaumont Mountain over 14 years (2004 to 2017).

| **Rank** | **Model structure** | **Df** | **logLik** | **AICc** | **ΔAIC** | **Weight1 (%)** | **Weight2 (%)** | **r^2^ (%)** |
| --- | --- | --- | --- | --- | --- | --- | --- | --- |
| 1 | S + P + B_2.1 + Y + S:Y + P:B_2.1 | 40 | -2197.872 | 4483.411 | 0.000 | 25.63 | 25.63 | 68.78 |
| 2 | S + P + B_2.1 + Y + S:Y | 39 | -2199.014 | 4483.632 | 0.221 | 22.95 | 48.58 | 68.52 |
| 3 | S + P + B_2.1 + Y + S:Y + S:B_2.1 | 42 | -2195.501 | 4483.645 | 0.234 | 22.80 | 71.39 | 68.53 |
| 4 | S + P + B_2.1 + Y + S:B_2.1 + P:B_2.1 + S:Y | 43 | -2194.543 | 4483.844 | 0.433 | 20.64 | 92.03 | 68.93 |
| 5 | S + P + B_2.1 + Y + S:P + P:B_2.1 + S:Y | 43 | -2196.813 | 4488.264 | 4.853 | 2.26 | 94.29 | 68.64 |
| 6 | S + P + B_2.1 + Y + S:P + S:B_2.1 + S:Y | 45 | -2194.473 | 4488.622 | 5.211 | 1.89 | 96.19 | 68.39 |
| 7 | S + P + B_2.1 + Y + S:Y + S:P | 42 | -2198.053 | 4488.631 | 5.220 | 1.88 | 98.07 | 68.39 |
| 8 | S + P + B_2.1 + Y + S:P + S:B_2.1 + P:B_2.1 + S:Y | 46 | -2193.434 | 4488.697 | 5.286 | 1.82 | 99.89 | 68.78 |
| 9 | S * P * B_2.1 + Y + S:Y | 49 | -2192.736 | 4494.377 | 10.966 | 0.11 | 100.00 | 68.53 |
| 10 | S + P + B_1.1 + Y + S:Y + P:B_1.1 | 39 | -2221.763 | 4529.831 | 46.419 | 0.00 | 100.00 | 63.90 |
| 11 | S + P + B_1.1 + Y + S:B_1.1 + P:B_1.1 + S:Y | 42 | -2220.213 | 4533.738 | 50.327 | 0.00 | 100.00 | 63.97 |
| 12 | S + P + B_1.1 + Y + S:P + P:B_1.1 + S:Y | 42 | -2220.971 | 4535.077 | 51.666 | 0.00 | 100.00 | 63.71 |
| 13 | S + P + B_1.1 + Y + S:P + S:B_1.1 + P:B_1.1 + S:Y | 45 | -2219.425 | 4539.096 | 55.685 | 0.00 | 100.00 | 63.79 |
| 14 | S * P * B_1.1 + Y + S:Y | 48 | -2218.794 | 4545.088 | 61.677 | 0.00 | 100.00 | 63.63 |
| 15 | S + P + B_2.1 + Y | 36 | -2233.588 | 4545.249 | 61.837 | 0.00 | 100.00 | 63.95 |
| 16 | S + P + B_2.2 + Y + S:Y + P:B_2.2 | 40 | -2229.972 | 4548.134 | 64.723 | 0.00 | 100.00 | 66.49 |
| 17 | S + P + B_2.2 + Y + S:B_2.2 + P:B_2.2 + S:Y | 43 | -2226.550 | 4548.244 | 64.833 | 0.00 | 100.00 | 66.53 |
| 18 | S + P + B_2.2 + Y + S:P + P:B_2.2 + S:Y | 43 | -2229.387 | 4554.082 | 70.671 | 0.00 | 100.00 | 66.32 |
| 19 | S + P + B_2.2 + Y + S:P + S:B_2.2 + P:B_2.2 + S:Y | 46 | -2225.907 | 4554.249 | 70.838 | 0.00 | 100.00 | 66.36 |
| 20 | S * P * B_2.1 + Y | 46 | -2228.177 | 4557.644 | 74.232 | 0.00 | 100.00 | 63.94 |
| 21 | S * P * B_2.2 + Y + S:Y | 49 | -2225.677 | 4560.966 | 77.554 | 0.00 | 100.00 | 66.16 |
| 22 | S + P + B_2.2 + Y + S:Y | 39 | -2256.692 | 4598.388 | 114.977 | 0.00 | 100.00 | 60.84 |
| 23 | S + P + B_2.2 + Y + S:Y + S:B_2.2 | 42 | -2253.504 | 4599.002 | 115.591 | 0.00 | 100.00 | 60.92 |
| 24 | S * P * B_1.1 + Y | 45 | -2250.125 | 4600.522 | 117.111 | 0.00 | 100.00 | 58.51 |
| 25 | S + P + B_2.2 + Y + S:Y + S:P | 42 | -2256.224 | 4604.346 | 120.935 | 0.00 | 100.00 | 60.45 |
| 26 | S + P + B_2.2 + Y + S:P + S:B_2.2 + S:Y | 45 | -2252.985 | 4604.915 | 121.504 | 0.00 | 100.00 | 60.44 |
| 27 | S * P * B_2.2 + Y | 46 | -2256.952 | 4616.116 | 132.704 | 0.00 | 100.00 | 61.46 |
| 28 | S + P + B_1.1 + Y + S:Y | 38 | -2277.791 | 4639.384 | 155.973 | 0.00 | 100.00 | 52.92 |
| 29 | S + P + B_1.1 + Y + S:Y + S:B_1.1 | 41 | -2277.049 | 4644.974 | 161.562 | 0.00 | 100.00 | 53.83 |
| 30 | S + P + B_1.1 + Y + S:Y + S:P | 41 | -2277.406 | 4645.536 | 162.124 | 0.00 | 100.00 | 52.56 |
| 31 | S + P + B_2.2 + Y | 36 | -2286.350 | 4650.536 | 167.125 | 0.00 | 100.00 | 57.50 |
| 32 | S + P + B_1.1 + Y + S:P + S:B_1.1 + S:Y | 44 | -2276.645 | 4651.085 | 167.674 | 0.00 | 100.00 | 53.48 |
| 33 | S + P + B_1.1 + Y | 35 | -2305.195 | 4687.123 | 203.712 | 0.00 | 100.00 | 49.40 |

*Notes:* The explanatory variables were elevation site (S), nymphal peak (P), beech masting index (B), and year (Y). There are three different beech masting indices, B[2,2], B[1,1], and B[2,1], which reflect the different time lags for the spring peak and fall peak. Interactions are indicated with a full colon; for example, the interaction between site and year is represented with ‘S:Y’. To model the bimodal phenology of the DON, a site-specific smoother function was applied to the calendar day, s(day, by = S). This site-specific smoother function is common to all the models and for simplicity is not shown in the model structure. The models are ranked according to their corrected Akaike information criterion (AICc). Shown for each model are the model rank (Rank), model structure, model degrees of freedom (Df), log-likelihood (logLik), AICc, difference in the AIC value from the top model (ΔAICc), model weight (Weight1), cumulative model weight (Weight2), and adjusted r-squared value (r^2^).

# SECTION S7 – Interpretation of the parameter estimates of the best model

In section S6, the best model of the monthly DON was base model 2 in **Table S2** (and model 2 in Table 5 in the main manuscript). The parameter estimates of this model are shown in Table 6 in the main manuscript. At each of the 4 elevation sites, the spring peak was always bigger than the fall peak (see **Figure 4** in the main manuscript). However, in the fixed effects structure of the best base model (model 2 in Table S2), the difference in intercepts between the fall and spring was positive (Fall – Spring contrast = 0.693, SE = 0.264, z = 2.629, p = 0.009), indicating that the fall peak is bigger than the spring peak. We believe that this counter-intuitive result is due to redundancy in the GAM between (1) the ‘nymphal peak’ factor in the fixed effects structure and (2) the site-specific smoother function of calendar day; these 2 components both model the spring and fall nymphal peaks.

The site-specific smoother function of calendar day models the bimodal phenology of the DON at each of the 4 elevation sites. In this smoother function, calendar days 1 to 243 represent the Spring nymphal peak and calendar days 244 to 365 represent the Fall nymphal peak. We also introduced the categorical factor ‘nymphal peak’ (2 levels: Spring and Fall) in the fixed effects structure. The motivation for this was to test for interactions between nymphal peak, elevation site, and the beech masting indices. However, the introduction of the nymphal peak in the fixed effects structure is redundant because the site-specific smoother of calendar day already accounts for differences in the DON between the spring and fall nymphal peaks, as well as for differences in phenology between the four elevation sites (**Figure S5**). In other words, the presence of the site-specific smoother of calendar day in the GAM makes it difficult for the fixed effects structure to detect the expected effect of the nymphal peak (i.e., a negative contrast between the fall and spring nymphal peaks).

Another criticism of the factor ‘nymphal peak’ is that this contrast between the Fall and Spring peak is non-sensical. The contrast is defined for a common date, day 0, which is the day before January 1 (i.e., 31 December). Thus, the interpretation of the parameter estimate for the nymphal peak is the difference in the DON between the fall and spring peaks on 31 December. This contrast is non-sensical because whatever date corresponds to day 0 cannot occur both in the fall and the spring. One potential solution to this problem would be to set to day 1 both the start of the spring peak (1 January) and the start of the fall peak (1 September). We tried this solution but the AICc values of the GAMs that used this scale for calendar day were much lower than the original calendar day scale.

To obtain a better understanding of how the scale of the calendar day in the smoother function influences the parameter estimates in the fixed effects structure, we decided to rescale the calendar day covariate. In the original model, calendar day was defined so that January 1 and December 31 corresponded to days 1 and 365, respectively. We created four other covariates where day 1 corresponded to March 1, June 1, September 1, and December 1, respectively. We re-ran the same 33 models in Table S2 for each of these 5 different scales of calendar day. For each of these 5 different scales, the best model was the same as in **Table S2**. The parameter estimates for the fixed effects were generally the same among the best models based on the 5 different scales. The exception was the parameter estimate for nymphal peak, which is the difference in intercepts between the Spring and Fall peak (**Table S3**). The contrast was either positive or negative depending on the scale of the calendar day (**Table S3**). The statistical significance also depended on the scale of the calendar day (**Table S3**). In summary, this analysis shows that the parameter estimate for the fixed factor nymphal peak is highly sensitive to the scale of the calendar day. This result suggests that the parameter estimate for nymphal peak in the fixed effects structure has limited meaning.

Table S3. Parameter estimate for the contrast between fall and spring for the best model using 5 different scales of the calendar day.

| **Scale** | **Day 0** | **Day 365** | **Contrast** | **Estimate** | **SE** | **z value** | **P** |
| --- | --- | --- | --- | --- | --- | --- | --- |
| 1 | 01-Jan | 31-Dec | Fall - Spring | 0.693 | 0.264 | 2.629 | 0.009 |
| 2 | 01-Mar | 28-Feb | Fall - Spring | -0.087 | 0.253 | -0.342 | 0.732 |
| 3 | 01-Jun | 30-May | Fall - Spring | 0.017 | 0.270 | 0.064 | 0.949 |
| 4 | 01-Sep | 31-Aug | Fall - Spring | -0.529 | 0.329 | -1.607 | 0.108 |
| 5 | 01-Dec | 30-Nov | Fall - Spring | 0.455 | 0.235 | 1.936 | 0.053 |

**Trade-off between fixed effects versus smoother function:** Given that the four elevation sites differ with respect to their phenology, we expected models with the interaction between elevation site and BM[2,1] index to have the highest support, but this was not the case (see **Table S2**). Again, the explanation is that the site-specific smoother function of calendar date captures the differences in phenology between the four elevation sites. To test this explanation, we re-ran our set of 33 models where the smoother function was the same for all four elevation sites (i.e., the smoother function was not site-specific). As expected, for this set of models, the best model was the same as before except that it included the interaction between elevation site and nymphal peak. This result demonstrates that there is a trade-off in complexity between the fixed effects and the smoother function. If the smoother function includes site-specific differences in phenology, we will not detect these site-specific differences in the fixed effects structure. Conversely, we will detect site-specific differences in phenology in the fixed effects structure if the smoother function does not account for these site-specific differences.

# SECTION S8 – AIC-based model selection of climate variables

**AIC-based model selection of climate variable 1:** Using base model 2 in **Table S2** as a starting point, we wanted to test which of the 66 climate variables explained additional variation in the DON over the 14 years of the study. The 66 climate variables included 3 field-measured climate variables on the day of tick sampling, 15 annual means from the weather stations (5 climate variables * 3 time lags), and 48 seasonal means from the weather stations (4 climate variables * 4 seasons * 3 time lags). We compared a set of 66 models; each model included base model 2 and one of the 66 climate variables. We used 3 different smoother functions: no interaction, interaction with nymphal peak, and interaction with elevation site. Thus, we investigated a total of 198 models (66 climate variables x 3 smoother functions = 198 combinations).

According to AIC-based model selection, the best model contained a smoother function stratified by nymphal peak of the field-measured temperature on the day of tick sampling (model 1 in **Table S4**). This best model had an AIC score of 4367.561, 93.8% of the support, an r^2^ value of 71.6%, and it explained 81.8% of the deviance (model 1 in **Table S4**). The AICc value of this best model (4367.561; model 1 in **Table S4**) was 116.071 units lower than the AICc value of the best base model (4483.632; model 2 in **Table S2**).

Table S4. Model selection results are shown for the first climate variable.

| **Rank** | **Climate variable 1** | **Df** | **logLik** | **AICc** | **ΔAIC** | **Weight1 (%)** | **Weight2 (%)** | **r^2^ (%)** |
| --- | --- | --- | --- | --- | --- | --- | --- | --- |
| 1 | s(fieldT, by = Peak) | 48 | -2130.494 | 4367.561 | 0.000 | 93.8 | 93.8 | 71.6 |
| 2 | s(fieldT) | 44 | -2138.499 | 4372.994 | 5.433 | 6.2 | 100.0 | 70.4 |
| 3 | s(fieldT, by = Site) | 57 | -2131.343 | 4390.969 | 23.408 | 0.0 | 100.0 | 69.8 |
| 4 | s(climapSnow_1, by = Site) | 50 | -2166.700 | 4444.039 | 76.478 | 0.0 | 100.0 | 70.6 |
| 5 | s(climapPR.W2, by = Site) | 55 | -2161.719 | 4446.476 | 78.915 | 0.0 | 100.0 | 70.3 |
| 6 | s(climapSD.S0, by = Site) | 51 | -2167.770 | 4448.301 | 80.740 | 0.0 | 100.0 | 73.9 |
| 7 | s(climapPR.L1, by = Site) | 54 | -2164.588 | 4450.224 | 82.663 | 0.0 | 100.0 | 71.2 |
| 8 | s(climapSD.S2, by = Peak) | 51 | -2168.937 | 4450.675 | 83.114 | 0.0 | 100.0 | 71.7 |
| 9 | s(climapPR.F2, by = Peak) | 51 | -2168.992 | 4450.932 | 83.371 | 0.0 | 100.0 | 74.0 |
| 10 | s(climapSD.F2, by = Peak) | 51 | -2168.833 | 4451.717 | 84.156 | 0.0 | 100.0 | 70.3 |
| 11 | s(climapRH.S0, by = Site) | 52 | -2168.005 | 4452.202 | 84.641 | 0.0 | 100.0 | 71.9 |
| 12 | s(climapSD.S0, by = Peak) | 45 | -2176.721 | 4452.347 | 84.786 | 0.0 | 100.0 | 71.3 |
| 13 | s(climapSD.S2, by = Site) | 48 | -2173.021 | 4453.341 | 85.779 | 0.0 | 100.0 | 73.1 |
| 14 | s(climapSD.W0, by = Peak) | 52 | -2168.850 | 4454.383 | 86.821 | 0.0 | 100.0 | 70.2 |
| 15 | s(climapPR.F2, by = Site) | 49 | -2172.770 | 4454.656 | 87.095 | 0.0 | 100.0 | 72.0 |
| 16 | s(fieldSD) | 45 | -2178.026 | 4454.664 | 87.103 | 0.0 | 100.0 | 69.4 |
| 17 | s(climapRH_2, by = Site) | 53 | -2168.757 | 4455.725 | 88.164 | 0.0 | 100.0 | 72.7 |
| 18 | s(climapRH.S0, by = Peak) | 45 | -2178.829 | 4456.292 | 88.731 | 0.0 | 100.0 | 72.0 |
| 19 | s(fieldSD, by = Peak) | 46 | -2177.864 | 4456.594 | 89.033 | 0.0 | 100.0 | 69.6 |
| 20 | s(climapT.S0, by = Peak) | 50 | -2173.210 | 4457.769 | 90.207 | 0.0 | 100.0 | 74.3 |

*Notes:* We investigated 66 different climate variables in combination with 3 different smoother functions (no interaction, interaction with nymphal peak, and interaction with elevation site) for a total of 198 models. Each model contained base model 2 from **Table S2**. Of the 198 models investigated, only the top 20 models are shown. The acronyms for the climate variables are given in Table 2 of the main manuscript. The prefixes ‘field’ or ‘climap’ refer to whether the climate variables were measured in the field or obtained from weather stations. The models are ranked according to their corrected Akaike information criterion (AICc). Shown for each model are the model rank (Rank), model structure, model degrees of freedom (Df), log-likelihood (logLik), AICc, difference in the AIC value from the top model (ΔAICc), model weight (Weight1), cumulative model weight (Weight2), and adjusted r-squared value (r^2^).

**AIC-based model selection of climate variable 2:** In the first round of model selection, the best model contained a smoother function stratified by nymphal peak of the field-measured temperature (model 1 in **Table S4**). Using this model as a starting point, we conducted a second round of model selection to determine whether any of the remaining 65 climate variables explained additional variation in the monthly DON. We compared a set of 65 models; each model included model 1 in **Table S4** and one of the remaining 65 climate variables. We used 3 different smoother functions: no interaction, interaction with nymphal peak, and interaction with elevation site. Thus, we investigated a total of 195 models (65 climate variables x 3 smoother functions = 195 combinations).

According to AIC-based model selection, the best model contained a smoother function stratified by elevation site of the snowpack in year *y*-1 (model 1 in **Table S5**). This best model had an AICc score of 4319.583, 75.053% of the support, an r^2^ value of 73.825%, and it explained 84.2% of the deviance (model 1 in **Table S5**). The AICc value of the best model with 2 climate variables (4319.583; model 1 in **Table S5**) was 47.961 units lower than the AICc value of the best model with 1 climate variable (4367.561; model 1 in **Table S4**) and it was 164.032 units lower than the AICc value of the best base model (4483.632; model 2 in **Table S2**).

After three rounds of model selection, which identified the base model, climate variable 1, and climate variable 2, the structure of the best model was as follows. The fixed effects contained elevation site, nymphal peak, beech masting index (BM[2,1]), year, and the interaction between site and year. There were 3 smoother functions: a smoother of calendar day stratified by elevation site, a smoother of the field-measured temperature stratified by nymphal peak, and a smoother of the snowpack in year *y*-1 stratified by elevation site.

In the three rounds of model selection, we tested a total of 426 models: 33 base models, 198 models for climate variable 1 and 195 models for climate variable 2. Investigating all possible combinations of the models in these three rounds of model selection would involve testing 1,274,130 models (33 x 198 x 195). Thus, an important advantage of sequential model selection over combinatorial model selection is that it allows the user to greatly reduce the number of models that must be tested (426 versus 1,274,130). An important disadvantage of sequential model selection is that it misses many potentially better models.

Table S5. Model selection results are shown for the second climate variable.

| **Rank** | **Climate variable 2** | **Df** | **logLik** | **AICc** | **ΔAIC** | **Weight1 (%)** | **Weight2 (%)** | **r2 (%)** |
| --- | --- | --- | --- | --- | --- | --- | --- | --- |
| 1 | s(climapSnow_1, by = Site) | 59 | -2093.030 | 4319.583 | 0.000 | 75.053 | 75.053 | 73.825 |
| 2 | s(climapSD.S0, by = Site) | 59 | -2094.399 | 4322.544 | 2.961 | 17.076 | 92.129 | 76.076 |
| 3 | s(climapPR.W2, by = Site) | 69 | -2083.600 | 4325.095 | 5.512 | 4.768 | 96.897 | 73.795 |
| 4 | s(climapSD.W0, by = Peak) | 62 | -2093.558 | 4327.075 | 7.492 | 1.772 | 98.670 | 73.769 |
| 5 | s(climapPR.L1, by = Site) | 63 | -2092.884 | 4330.188 | 10.605 | 0.374 | 99.043 | 73.963 |
| 6 | s(climapSD.F2, by = Peak) | 61 | -2096.506 | 4330.685 | 11.102 | 0.291 | 99.335 | 73.729 |
| 7 | s(climapRH_2, by = Site) | 59 | -2099.037 | 4331.081 | 11.498 | 0.239 | 99.574 | 76.181 |
| 8 | s(climapSD.W0, by = Site) | 64 | -2092.852 | 4332.445 | 12.862 | 0.121 | 99.695 | 74.082 |
| 9 | s(climapT.S0, by = Site) | 61 | -2097.617 | 4333.340 | 13.757 | 0.077 | 99.772 | 76.379 |
| 10 | s(climapRH.L1, by = Site) | 69 | -2088.340 | 4334.594 | 15.011 | 0.041 | 99.813 | 73.310 |
| 11 | s(climapPR.F2, by = Peak) | 60 | -2098.979 | 4334.810 | 15.227 | 0.037 | 99.851 | 76.836 |
| 12 | s(climapRH.W0.S0, by = Site) | 66 | -2091.673 | 4335.772 | 16.189 | 0.023 | 99.873 | 72.616 |
| 13 | s(climapT.W2, by = Site) | 60 | -2100.462 | 4335.815 | 16.232 | 0.022 | 99.896 | 72.189 |
| 14 | s(climapT.W0, by = Site) | 68 | -2090.261 | 4336.081 | 16.498 | 0.020 | 99.915 | 77.036 |
| 15 | s(climapRH.S0, by = Site) | 63 | -2095.802 | 4336.380 | 16.797 | 0.017 | 99.932 | 74.276 |
| 16 | s(climapT.F2, by = Site) | 65 | -2094.119 | 4336.545 | 16.962 | 0.016 | 99.948 | 74.984 |
| 17 | s(climapT.W2) | 54 | -2108.128 | 4336.799 | 17.216 | 0.014 | 99.962 | 70.788 |
| 18 | s(climapSD.F2) | 56 | -2106.319 | 4338.777 | 19.194 | 0.005 | 99.967 | 71.614 |
| 19 | s(climapT.S0) | 55 | -2107.426 | 4339.424 | 19.841 | 0.004 | 99.970 | 77.092 |
| 20 | s(climapSD.L2, by = Site) | 61 | -2100.553 | 4339.693 | 20.110 | 0.003 | 99.974 | 76.816 |

*Notes:* We investigated 65 different climate variables in combination with 3 different smoother functions (no interaction, interaction with nymphal peak, and interaction with elevation site) for a total of 195 models. Each model contained model 1 from **Table S4**. Of the 195 models investigated, only the top 20 models are shown. The acronyms for the climate variables are given in Table 2 of the main manuscript. The prefixes ‘field’ or ‘climap’ refer to whether the climate variables were measured in the field or obtained from weather stations. The models are ranked according to their corrected Akaike information criterion (AICc). Shown for each model are the model rank (Rank), model structure, model degrees of freedom (Df), log-likelihood (logLik), AICc, difference in the AIC value from the top model (ΔAICc), model weight (Weight1), cumulative model weight (Weight2), and adjusted r-squared value (r^2^).

# SECTION S9 – Comparison of the observed versus the predicted values of the DON over the 14-year study period for each of the 4 elevation sites


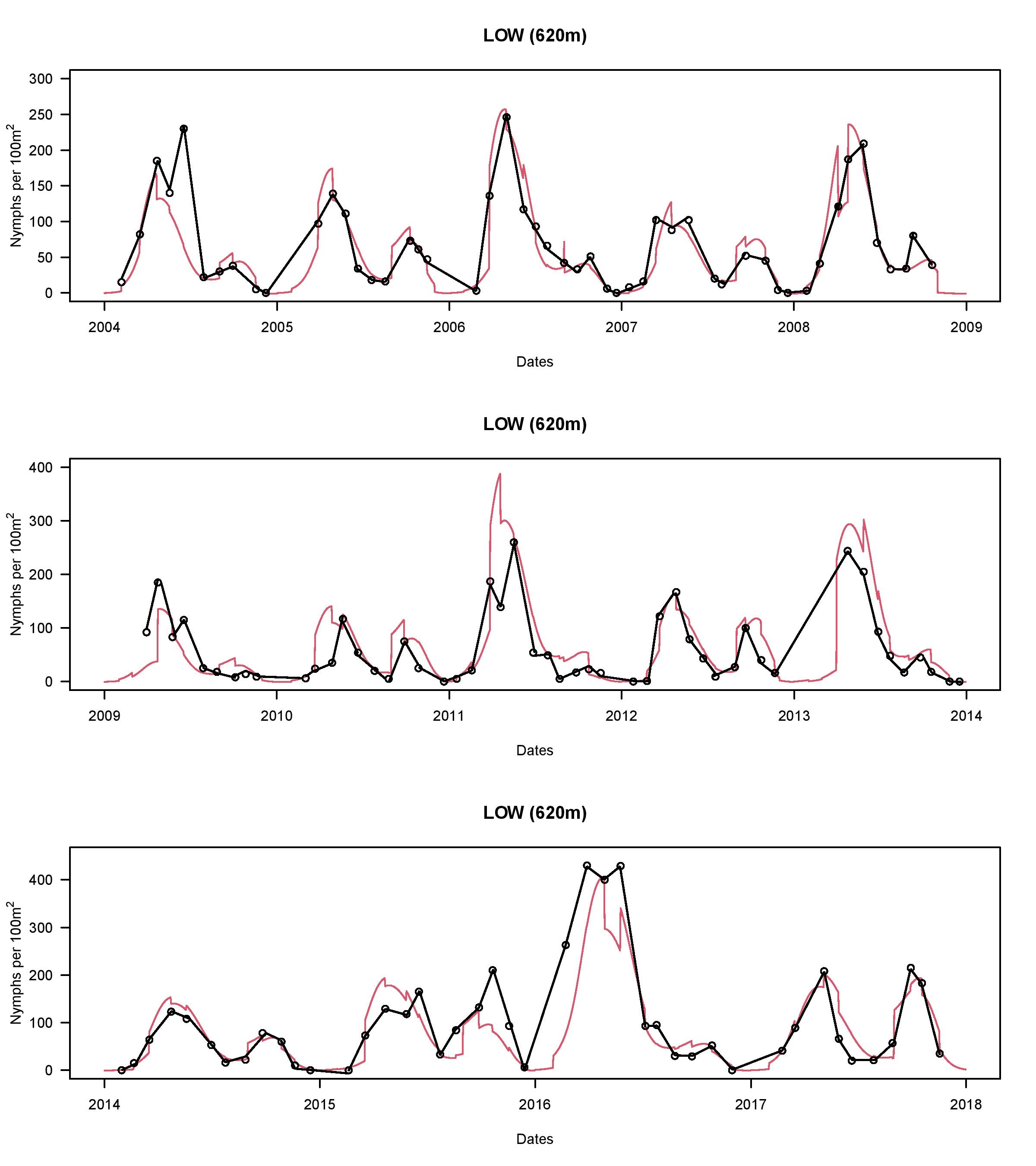


Figure S6. Comparison of the observed values of the DON versus the predicted values of the DON at the low site for the 14-year study period. This time series graph uses the best model (model 1 in Tables S5). The black line represents the observed values, and the red line represents the predicted values.


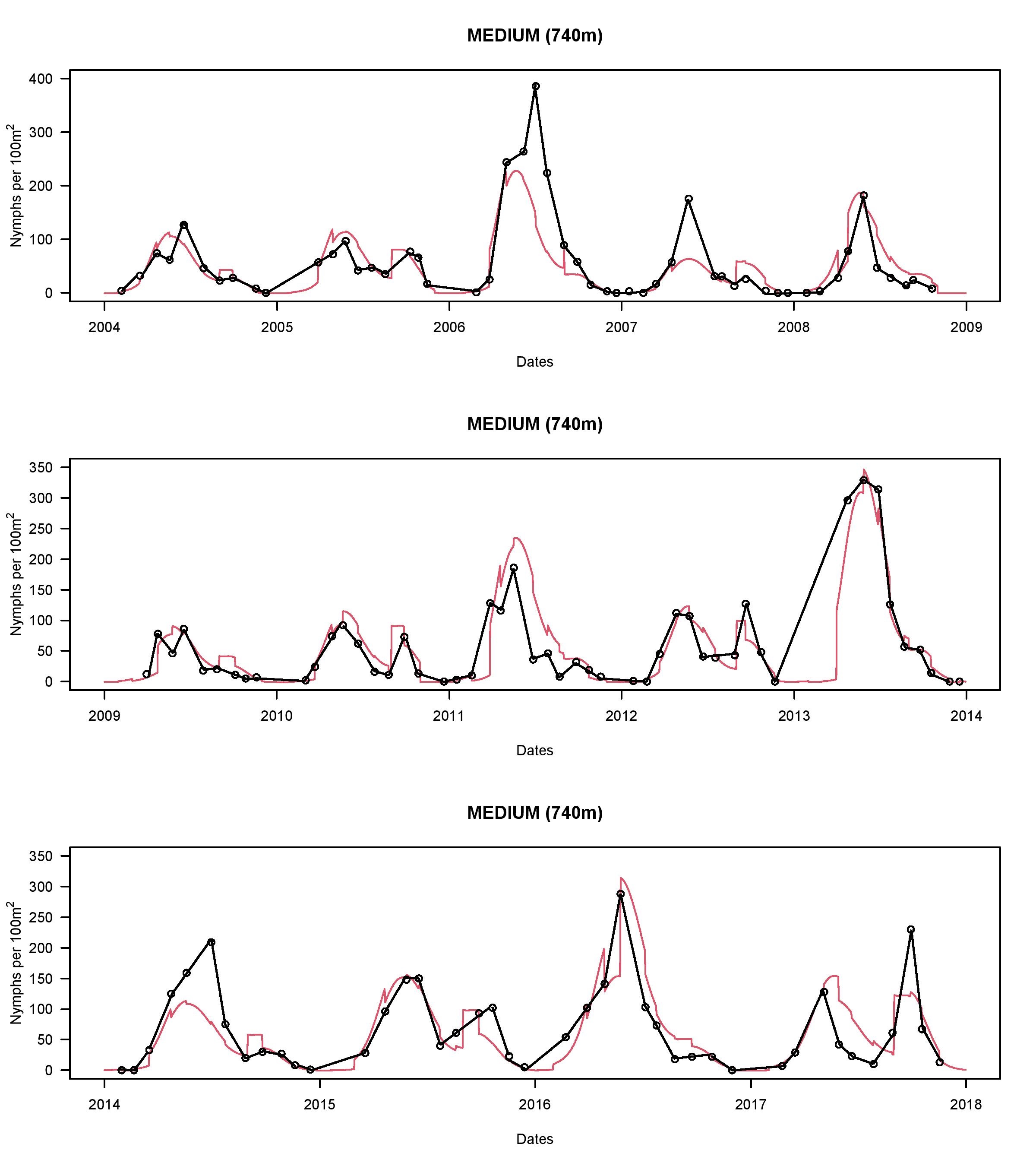


Figure S7. Comparison of the observed values of the DON versus the predicted values of the DON at the medium site for the 14-year study period. This time series graph uses the best model (model 1 in Tables S5). The black line represents the observed values, and the red line represents the predicted values.


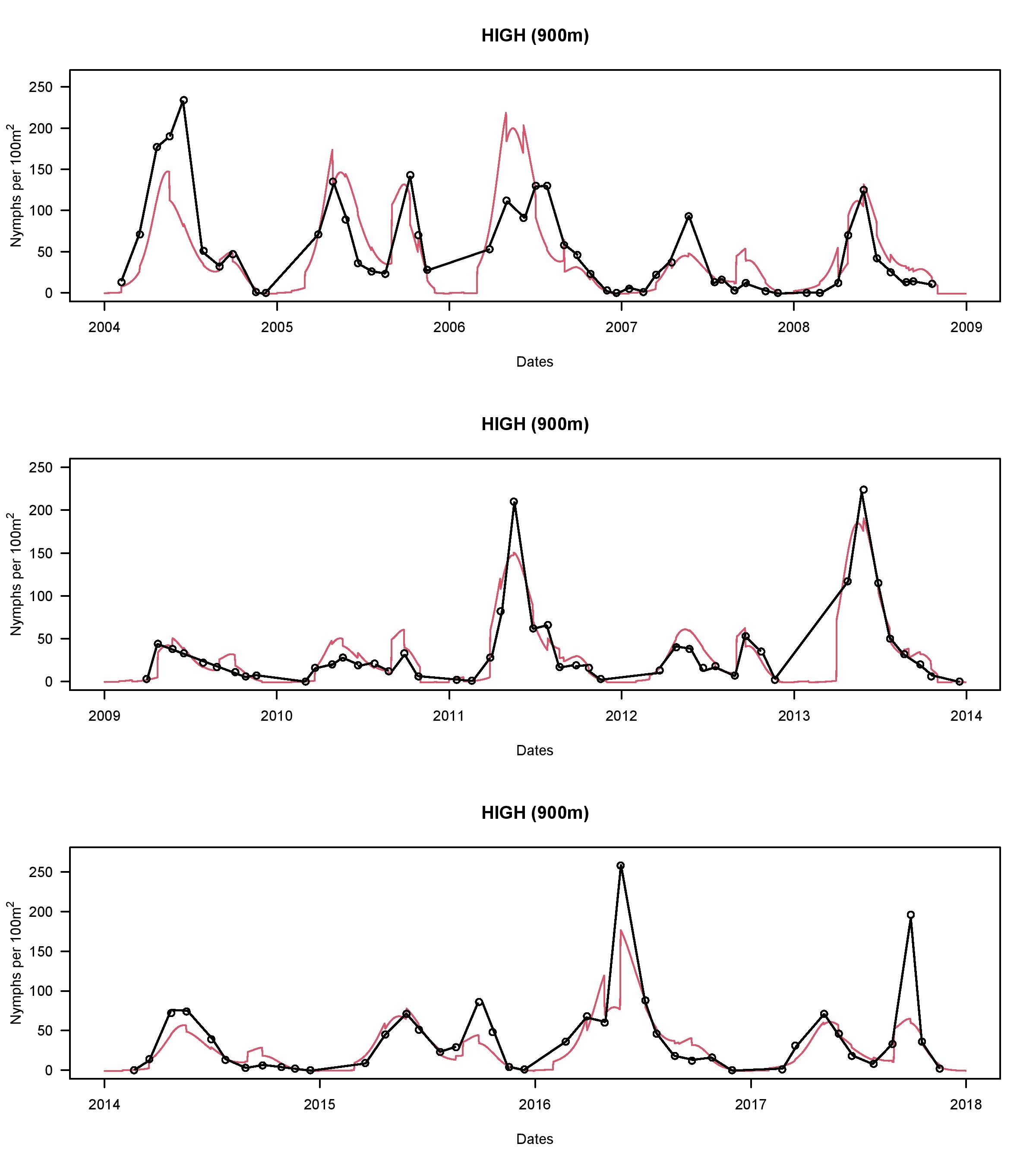


Figure S8. Comparison of the observed values of the DON versus the predicted values of the DON at the high site for the 14-year study period. This time series graph uses the best model (model 1 in Tables S5). The black line represents the observed values, and the red line represents the predicted values.


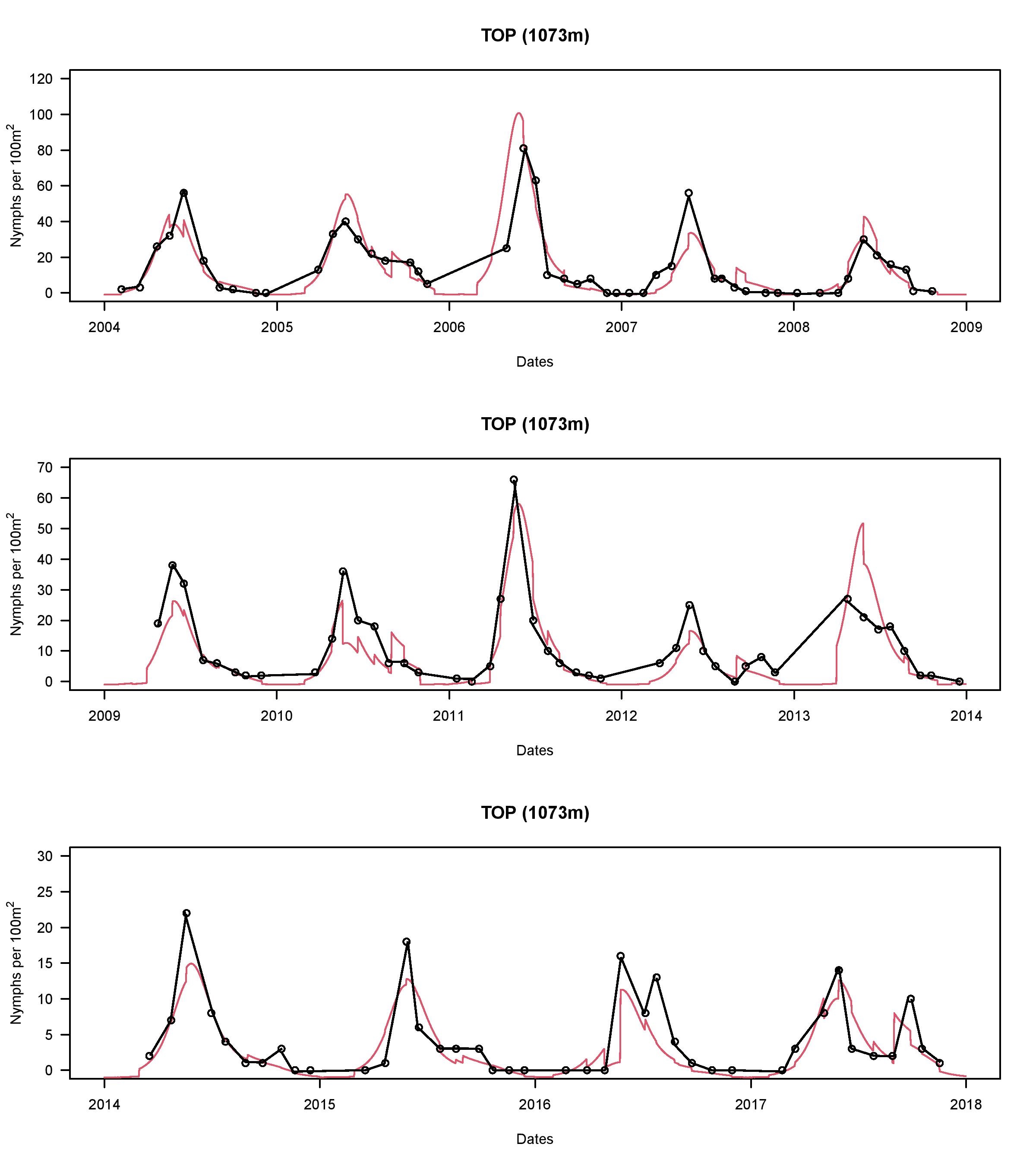


Figure S9. Comparison of the observed values of the DON versus the predicted values of the DON at the top site for the 14-year study period. This time series graph uses the best model (model 1 in Tables S5). The black line represents the observed values, and the red line represents the predicted values.

# SECTION S10 – Auto-correlation of the residuals

The monthly DON values over the 15 years of the study represent a time series. Time series data often contain autocorrelations because datapoints that are close together in time are more likely to have similar values. If this autocorrelation in a time series is not properly modelled, the residuals can also show autocorrelation, which violates the assumption that the residuals are independent. We used the acf() function in the TSA package to calculate the auto-correlation among the residuals of the best model (model 1 in Table S5) for a range of time lags. Figure S10 shows the correlation coefficient between the residuals that were separated by 0, 1, 2, …, and 27 months. The correlations between residuals separated by 1, 2, and 3 months were 0.316, 0.116, and 0.015, respectively, indicating that autocorrelation between residuals disappears after 2 months. After excluding the correlation with a time lag of 0, only 3 of 27 correlation coefficients had an absolute value > 0.10. These 3 correlations occurred with time lags 1, 2, and 10 months and had coefficients of 0.316, 0.116, and -0.151. The main purpose of our study was to investigate whether inter-annual variation in the fall peak was influenced by beech masting with a 2-year or a 1-year time lag (i.e., 24 months versus 12 months). In Figure S10, the autocorrelation between residuals is essentially non-existent after 1 month. This weak autocorrelation among adjacent months is unlikely to influence our main conclusions about the origin of the fall peak, which is explained by much longer time lags (i.e., 24 months versus 12 months).

Figure S10. Correlation coefficient of the residuals is shown for 27 different time lags. The correlation of residuals with themselves (time lag = 0) is perfect, and the correlation coefficient (r) is 1.000. The correlation coefficients for the residuals with a time lag of 1, 2, and 3 months are 0.316, 0.116, and 0.015, respectively. This result suggests that autocorrelation between residuals is essentially non-existent after 1 month.

References

Bregnard, C., O. Rais, and M. J. Voordouw. 2020. Climate and tree seed production predict the abundance of the European Lyme disease vector over a 15-year period. Parasit Vectors **13**:408.

Bregnard, C., O. Rais, and M. J. Voordouw. 2021. Masting by beech trees predicts the risk of Lyme disease. Parasites & Vectors **14**:168.
